# Correlates of social role and conflict severity in wild vervet monkey agonistic screams

**DOI:** 10.1371/journal.pone.0214640

**Published:** 2019-05-01

**Authors:** Stéphanie Mercier, Eloïse C. Déaux, Erica van de Waal, Axelle E. J. Bono, Klaus Zuberbühler

**Affiliations:** 1 Department of Comparative Cognition, Institute of Biology, University of Neuchâtel, Neuchâtel, Switzerland; 2 Inkawu Vervet Project, Mawana Game Reserve, KwaZulu-Natal, Vryheid, South Africa; 3 Department of Ecology and Evolution, University of Lausanne, Lausanne, Switzerland; 4 Anthropological Institute and Museum, University of Zürich, Zürich, Switzerland; 5 School of Psychology and Neuroscience, University of St Andrews, St Andrews, Scotland, United Kingdom; Universidade de São Paulo, BRAZIL

## Abstract

Screams are acoustically distinct, high-pitched and high-amplitude calls, produced by many social species. Despite a wide range of production contexts, screams are characterised by an acoustic structure that appears to serve in altering the behaviour of targeted receivers during agonistic encounters. In chimpanzees, this can be achieved by callers producing acoustic variants that correlate with their identity, social role, relationship with the targeted recipient, the composition of the audience and the nature of the event. Although vervet monkeys (*Chlorocebus pygerythrus*) have been studied for decades, not much is known about their agonistic screams. Here, we examined agonistic screams produced by wild vervet monkeys to investigate the degree to which caller identity, social role and conflict severity affected call structure. We found that screams were both individually distinctive and dependent of the agonistic events. In particular, victim screams were longer and higher-pitched than aggressor screams, while screams produced in severe conflicts (chases, physical contact) had higher entropy than those in mild conflicts. We discuss these findings in terms of their evolutionary significance and suggest that acoustic variation might serve to reduce the aggression level of opponents, while simultaneously attracting potential helpers.

## Introduction

Many animal species produce acoustically distinct screams in a range of contexts. These calls are usually high-pitched and amongst the highest amplitude vocalisations of a species’ vocal repertoire (e.g. *Macaca spp*. [1]). As a result, screams often travel over considerable distances to serve different functions, such as repelling aggressors (e.g. tree shrew, *Tupaia belangeri* [2]) or predators (e.g. European starling, *Sturnus vulgaris* [3]), recruiting allies (e.g. pigtail macaque, *Macaca nemestrina* [4]), advertising copulations (e.g. New Guinea singing dog, *Canis hallstromi* [5]) or facilitating group fusions (e.g. dwarf mongoose, *Helogale parvula* [6]). Despite their universal structure, screams often possess substantial between-context acoustic variation, particularly with regards to duration, pitch and proportion of tonal and noisy components [7, 8].

A number of theories have been put forward to explain patterns of acoustic variation in animal calls. First, Morton’s motivational-structural rules [9] state that vocalisations emitted during hostile and aggressive situations tend to be low pitched, harsh sounds with a broadband frequency range (large inter-quartile range and high entropy), with harshness being positively related to non-linear acoustic phenomena (NLP [10]). In contrast, calls produced in friendly interactions or when a caller is fearful tend to be tonal and high pitched [9]. Similar arguments have been made for differences in urgency with signallers producing longer calls at higher rates in urgent contexts compared to other situations [11].

Second, Owren & Rendall [12, 13] suggested that the primary function of vocal signals is to directly influence the behaviour of receivers through specific acoustic features. For example, chaotic spectral sounds with sharp onsets and dramatic fluctuations in both frequency and amplitude are likely to increase attention levels in receivers, and may become aversive when repetitive [13, 14]. To humans, such acoustic structures tend to be intrinsically ‘unpleasant’ [15], suggesting the same may be the case for animals [13]. Another important feature of animal calls are non-linear phenomena (NLP [10]), which include frequency jumps, bi-phonation, sub-harmonics and deterministic chaos (see [16] for further descriptions). While sub-harmonics and chaotic segments appear to prevent habituation [17], bi-phonation could signal a caller’s physical condition (e.g. chimpanzees, *Pan troglodytes schweinfurthii*, pant hoots [18]) and facilitate individual recognition (e.g. dholes, *Cuon alpinus*, yap-squeaks [19]).

Third, Briefer [20] argued that vocalisations might be good indicators of emotional states in animals, where emotion is defined in terms of valence and arousal [21]. Valence (whether a situation is perceived as positive or negative) is thought to mainly be linked to energy distribution and frequency spectrum, while arousal (reflecting situational intensity) is thought to be linked to fundamental frequency, duration and calling rate, produced by differences in respiration [20]. More specifically, calls produced in situations of high arousal should therefore be high frequency, high amplitude, noisy, long and produced at high rates.

Such theories provide mechanistic explanations of acoustic structures that ultimately relate to a call’s function. With regards to agonistic screams, putative functions include repelling opponents via acoustically obnoxious call characteristics [12, 22] and attracting helpers [23]. Interfering in ongoing aggression is likely to be very costly for bystanders, suggesting that listeners may require specific information before deciding whether to intervene or not [24]. In particular, acoustic variation should provide reliable identity cues and describe the ongoing event in sufficient details so that potential third-party helpers can intervene only when beneficial, such as to support kin or allies that are involved in severe fights. Indeed, several studies on a range of species have shown that screams differ according to caller identity, including, for example, European starlings [25], white-faced capuchins, *Cebus capucinus* [26] or vampire bats, *Phyllostomidae* family [27]. Crucially, playback experiments in several primate species have demonstrated that listeners adjust their support according to the identity of the caller, suggesting that they attend to individually distinct features of the calls (e.g., squirrel monkey, *Saimiri sciureus* [28], Japanese macaque, *Macaca fuscata* [29] or Barbary macaque, *Macaca sylvanus* [30]). For example, rhesus monkeys, *Macaca mulatta*, respond more strongly to playbacks of screams by kin than unrelated individuals [31].

Similarly, there is evidence in some primates that screams convey features of the ongoing aggressive interaction that may be essential for bystanders, such as the caller’s social role (aggressor vs. victim) and the severity of the conflict (mild vs. severe). For example, Geoffroy’s spider monkeys, *Ateles geoffroyi*, produce screams with lower fundamental frequencies as aggressors than as victims as well as during severe than mild conflicts [32]. In chimpanzees, screams also differ in duration and spectral structure according to the social role of signallers [33] and receivers adapt their support according to whether victims are involved in mild or severe conflicts [24]. Moreover, there is evidence that nearby listeners can react appropriately according to the nature of agonistic events, by attending to a combination of acoustic cues and their general social knowledge [34].

Like other primates, vervet monkeys often produce screams in a variety of contexts, particularly during social conflicts ([35]; S1 Appendix). Screams can be accompanied by facial expressions that range from partially closed to widely open months with exposed teeth [35], probably reflecting how conflict severity is perceived. Using playback experiments, Hauser [36] argued that adult listeners were able to attend to identity cues present in infant screams. In further experiments, females discriminated between screams of their own and unrelated juveniles, with bystander females looking towards the mothers of call providers, suggesting third-party knowledge of mother-offspring dyads [37]. Whether the acoustic structure of vervet monkey screams differs according to the social role of signallers and conflict severity has, to our knowledge, not yet been addressed.

The aim of this study was to describe the structure of all high-pitched calls produced by wild vervet monkeys during agonistic interactions. We selected 15 common acoustic parameters for which we had predictions on how they should be affected according to the previously described theories [9, 10, 12, 13, 20], especially concerning the social role of signallers (aggressor vs. victim) and conflict severity (mild vs. severe). First, we expected screams produced by victims to be higher pitched and to contain more NLP than the ones produced by aggressors. Second, we predicted that victims of mild aggression would emit shorter, higher-pitched, and more tonal screams given at lower rates than screams produced by victims of severe aggression (see S2 Appendix for detailed predictions). As identity cues are important, especially if calls are directed at third-party bystanders, we investigated whether screams were individually distinct and, crucially, whether the social role of signallers and/or conflict severity affected the rate of support obtained by signallers.

## Methods

### Ethical note

All animals were fully habituated to the presence of human observers and generally ignored researchers during their daily activities. We used standard ways of collecting natural behavioural data and received approval by Ezemvelo KZN Wildlife, the governmental organization in charge of Kwa-Zulu Natal wildlife conservation and biodiversity, and the University of Cape Town, South Africa. Furthermore, protocols of feeding experiments ran by other researchers during data collection were also approved by Ezemvelo KZN Wildlife and by Ethics Committee of the School of Psychology and Neuroscience, University of St Andrews.

### Study site and species

The study took place in the Savannah biome of the Mawana Game Reserve, a private farm of 12,000 hectares in KwaZulu-Natal, South Africa (S28°00.327; E031°12.348), base of the Inkawu Vervet Project (IVP). Subjects were 26 individuals from one group of wild vervet monkeys: Baie Dankie (BD; eight adult females, seven juvenile females and 11 juvenile males, see S3 Appendix for detailed descriptions of call providers). Although group size varied over time due to births, deaths and migrations, the group contained multiple males (defined as adults after their first migratory event, but excluded from analyses as they rarely scream), females (defined as adults after they gave birth for the first time) and juveniles.

### Data collection

We recorded scream vocalisations over four years (17.07.12–06.11.2015, see S3 Appendix for detailed number of calls, bouts and events used for analyses). Recordings were made with a Marantz PMD661 (sampling rate of 44.1 kHz, resolution 24 bits) and a Sennheiser MKH416 microphone and stored as wav files. Recordings were based on ad libitum sampling, that is, any call observed during natural conflicts or during conflicts following food provisioning experiments carried out as part of other research, using four different experimental methods (‘box’ experiment: subjects had to retrieve food from a closed container [38], ‘jingle’ experiment: conditioned subjects were rewarded following individualised acoustic cues [39], ‘corn’ experiment: large plastic containers with corn were provided for the entire group to feed on [40, 41], ‘vervetable’ experiment: subjects had to copy a demonstrator’s object manipulations to access a small amount of food [42]). Conflicts occurred in all four experimental conditions.

For any conflict, we recorded the context (observation vs. experiment), caller and recipient identity and behaviour (feeding vs. other), social role (aggressor vs. victim), conflict severity (mild vs. severe), any third-party interventions (yes vs. no), GPS location, weather condition, audience size (i.e. number of individuals present within 10m of the interaction) and, if possible, audience identity.

### Definitions

#### Screams

We defined a scream as any call that was high-pitched, shrill-sounding and produced during an agonistic event (see S1 Appendix for scream production in vervet monkeys and S4 Appendix for details on acoustic data). Although the mean scream intervals in our dataset was 0.5s (measured from spectrograms created in Praat version 5.4.13 [43], www.praat.org), we coded a scream as a distinct utterance if it was separated by at least 0.3s of silence from another scream. To reduce the problem of non-independency of data, we selected calls from different bouts or events whenever possible (see S3 Appendix for detailed information of the number of events, bouts and screams used for each call provider). We excluded all aggressive calls, such as barks, grunts and any unclassifiable vocalisations [35].

#### Conflict

We defined conflict as an agonistic interaction that started when an individual approached another one in a threatening way, i.e., performing at least one of the aggressive behaviours described in Table 1, and lasted until both opponents resumed normal activities. While such events sometimes started without specific signalling (e.g., one individual displacing another one silently), we measured event duration from the onset of the first and offset of the last screams produced during the event using oscillograms and spectrograms created in Praat [43] using Fast Fourier Transformations (Hanning window shape, window length = 0.01s, number of time steps = 1000, number of frequency steps = 500 and dynamic range = 40dB). We classified events as separate from each other if there was a change in partner identities or if there were separated by an interval of at least 30s without any agonistic behaviour, in a similar way as screaming bouts were separated in chimpanzees [33]. Screams were typically emitted in bouts, which we defined as different stages of the conflict distinct from each other when either the social role of signallers or the severity of the conflict changed (S4 Appendix).

**Table 1:** Description of the aggressive behaviours determining conflict severity (modified from Slocombe and Zuberbühler [44]).

Aggression	Conflict	Risk of injury	Aggressive behaviour
Mild	Non-directed	Low	Approach, aggression calls, monopolise
Mild	Directed	Low	Stare, attack, displace
Severe	Chase	High	Chase
Severe	Physical contact	High	Hit, grab, bite

#### Conflict severity

Similarly to the study on wild chimpanzee screams [44], we distinguished two types of agonistic interactions according to conflict severity. We considered a conflict being of mild aggression if the risk of injuries resulting from the conflict was low, while a severe aggression could generate potential harm either through direct physical contact or through accidental injuries, resulting from an escaping or chasing behaviour (Table 1). While some behaviours were clearly targeted at specific opponents (e.g., stare, chase, physical contact), others were not directed but provoked reactions in nearby conspecifics, such as approaching a feeding spot, which could trigger screams from feeding individuals.

#### Social role of signallers

During agonistic interactions, individuals could take two basic social roles: aggressor or victim. We defined individuals as aggressors if they performed at least one of the following behaviours: stare, monopolise, attack, displace, chase, hit, grab, bite or produce aggressive calls. We classified individuals as victims if they performed at least one of the following behaviours: avoid, retreat, jump aside, crawl, flee, look for support or redirect the aggression on another individual, including humans (see S5 Appendix for a detailed ethogram). However, the social role of an individual might change during a single event, as for example when a victim being chased by an aggressor redirects the aggression towards a new victim, thus becomes an aggressor, or by recruiting support from bystanders that help the victim to chase away the initial aggressor. In such cases of role switching, we defined the different stages of conflicts as separate bouts (S4 Appendix).

#### Support

Support was defined as an individual entering an already ongoing conflict and behaving aggressively towards one of the two opponents. We recorded whether or not support occurred during each conflict (see S5 Appendix for a detailed ethogram). Whenever possible, we collected the identity of all animals involved.

### Acoustic analyses

We only subjected screams to acoustic analyses if they were of good quality (low background noise, no clipped sounds or reverberation noise) and if the required contextual information was available. We tried to match screams exchanged between the same two individuals during the same period (or with minimal time intervals). This was to control for potential effects of opponent identity or developmental effects. As adult males rarely produced screams, we excluded them from the analyses. Furthermore, we avoided problems of non-independency of data due to screams from different bouts of the same events (12.0% of samples, 45/374) by incorporating ‘events’ as a random factor in our statistical models.

Following visual inspection of the spectrograms, we excluded 53.8% of the samples (437/811) due to poor recording quality following the above criteria. The resulting dataset consisted of N = 374 screams from 119 bouts and 95 events, produced by 26 individuals of all age-sex classes, except adult males (see S3 Appendix for details and S4 Appendix for acoustic data). We selected 15 acoustic parameters to describe the screams’ acoustic properties and the temporal structure of their bouts (nine parameters at the call level and six at the bout level; Table 2). We chose commonly used parameters that allowed us to make clear predictions on scream variation according to the social role of signallers [33] and conflict severity (S2 Appendix; [32]). We were unable to use fundamental and formant frequencies due to the noisy acoustic components often present in vervet monkey screams. Temporal parameters were extracted from spectrograms and oscillograms created in Praat [43]. All other acoustic parameters were extracted from spectrograms and spectral slices (see S4 Appendix for examples) created with Seewave [45] and tuneR packages [46] in R version 1.0.143 [47] using the following settings: sampling rate 44.1 kHz, 16 bits accuracy, Fast Fourier Transformation with 512 samples, Hanning window and 90% overlap.

**Table 2:** Definitions of the 15 selected acoustic parameters.

Parameters	Definitions
CALL LEVEL	
Scream duration (s)	Duration of one scream, described as a continuous vocal unit along a time axis on the spectrogram that is not interrupted by more than 0.03s of silence
Peak frequency (kHz)	Frequency taken from the spectral slice at which maximum acoustic energy occurs in the entire scream
Coefficient of frequency variation *	Coefficient of frequency variation representing the range of frequency variation around the mean
Coefficient of frequency modulation	Coefficient of frequency modulation representing frequency changes over time
Absolute transition onset (Hz)	Frequency at which maximum acoustic energy occurs at the middle of the scream minus the one occurring at the beginning of the scream
Absolute transition offset (Hz)	Frequency at which maximum acoustic energy occurs at the end of the scream minus the one occurring at the middle of the scream
Frequency quartile 50 (Hz)	Frequency quartile that divides the scream into two frequency intervals of equal energy (corresponding to 50–50%)
Inter-quartile range (Hz)	Inter-quartile range representing the difference of the frequency quartile of 25% and 75% (corresponding to Q25-Q75)
Shannon entropy	Measure of the uniformity of the power spectrum, with white noise having an entropy value of 1 and pure tone having an entropy value of 0
BOUT LEVEL	
Bout duration (s) *	Duration of one bout, described as a specific stage of a conflict in which the social role of signallers and the conflict severity is stable
Number of screams	Total number of screams emitted by the signaller within a single bout
Average scream duration (s)	Average duration of all screams produced by one individual within a bout
Scream intervals (s) *	Average duration of the intervals between the screams produced by one individual within a bout
Scream rate (number of screams/s)	Rate of screams produced by one individual within a bout delivered per time unit
Percentage of screams with NLP (%)	Percentage of screams emitted in a bout containing at least one of the four following forms of NLP: frequency jumps, sub-harmonic segments, bi-phonation or chaotic segments

* Parameters excluded due to high correlations or failure to reach symmetrical distribution, leading to analyses using 12 acoustic parameters (eight at the call level and four at the bout level; S2 Appendix)

### Inter-observer reliability

We tested for inter-observer reliability of behavioural and acoustic data using Cohen’s Kappa method [48]. First, two observers coded behavioural sequences of agonistic interactions recorded in the field to obtain a proportion of agreement for the number of bouts within the conflict, the social role of signallers and conflict severity (SM-EW, Cohen’s Kappa, number of bouts: N = 50 i.e. 49% of all data, k = 0.88; social role: N = 63 i.e. 49% of all data, k = 0.90; severity: N = 63 i.e. 54% of all data, k = 0.87). Second, two observers annotated 20 raw recordings (i.e. 14% of all data, see S1 Appendix for an example of annotated recording) in Praat to then compare the resulting text grids allowing us to obtain a Cohen’s Kappa value for five acoustic parameters: duration of event (counted as an agreement if differences in measurement were <1s), total number of screams produced within a bout, average duration of screams produced in a bout (counted as an agreement if differences in measurement were <0.3s), proportion of screams of good quality and percentage of screams presenting at least one form of NLP (SM-ED, Cohen’s Kappa, N = 20 recordings, event duration: *k* = 1.00; number of screams: *k* = 0.88; scream duration: *k* = 0.84; analysable screams: *k* = 0.82; NLP: *k* = 0.70, see S6 Appendix for detailed protocol used to test for inter-observer reliability).

### Statistical analyses

#### Context and caller identity

We initially performed fully crossed permutated discriminant function analyses (pDFA) using an R script provided by R. Mundry to investigate whether context (i.e. screams recorded during natural follows vs. during feeding experiments) affected call and bout related acoustic parameters while controlling for individual variation [49]. We then tested whether screams were individually distinct using a discriminant function analysis (DFA). We used the jack-knifed method, which derives discriminant functions from a subset of the data (classification success) and uses those to classify the remaining observations (cross-validation success). We selected on average 51.7% calls per individuals from datasets to maintain a balanced training set, and obtained mean classification and cross-validation successes using 100 randomly selected samples. We evaluated the success of the procedures by comparing the success rates obtained to the success rates obtained on 1,000 permutated datasets, where calls were randomized across individuals. This comparative approach allows obtaining expected classification rates, which pertain directly to the dataset investigated, rather than relying on a theoretical distribution (see [50] for more details on the methods). Data were transformed to reach approximate symmetrical distribution when needed and scaled (mean = 0 and s.d. = 1). We checked multi-collinearity among variables using correlation matrices and highly correlated variables (i.e. when > 0.80) were excluded from pDFAs and DFAs. We excluded the coefficient of frequency variation variable as it was highly correlated to Q50, and bout duration as it was highly correlated to both the number of screams and screams rate. Furthermore, we excluded scream intervals as transforming this parameter did not help to reach symmetrical distribution, and due to its correlation with both bout duration and scream rate. For the DFAs on caller identity, we used a crossed design with fully balanced dataset, to control for the participation of each individual in social role and severity, which reduced dramatically our sample size (resulting in N = 4 and N = 8 individuals for caller identity at the call and bout levels respectively). We thus re-ran these analyses to check for the robustness of our analyses with an increased sample size using an incomplete crossed design (increasing to N = 23 individuals and N = 13 individuals for caller identity at the call and bout levels respectively).

#### Social role of signallers and conflict severity

To investigate whether the social role of the signaller and conflict severity influenced the acoustic characteristics of screams, we performed a series of linear mixed models fitted by restricted maximum likelihood (REML) with Laplace approximation, normal or lognormal distributions and logit-link function (LMER [51]). We used each acoustic parameter as the response variable (leading to 12 GLMMs) and three fixed effects: social role of signallers (binary: aggressor vs. victim), conflict severity (binary: mild vs. severe) and their interaction. We included caller identity as well as events as random effects to control for repeated measures, thus avoiding pseudo-replication [52]. We then checked for homogeneity of the data and the distribution of residuals using graphical analyses of residuals (using bwplots, density plots, qqplots and binned plots) and checked for influential individuals and outliers, removing them only if necessary (that is, if it helped to reach approximate symmetrical distribution and did not affect our results). Consequently, we removed a total of six outliers and one influential individual (one outlier for the coefficient of frequency modulation, one outlier for the absolute transition offset, one influential individual (Alsi) for the frequency at quartile 50, one outlier for the Shannon entropy, one outlier for the number of screams and two outliers for the percentage of NLP).

#### Support

As one suggested function of agonistic screams is to recruit support, we used a generalized linear mixed model [53] fitted with a binomial structure and logit-link function to examine the influence of the social role of signallers and conflict severity on the occurrence of support during conflicts. We used the occurrence of support as the response variable, that is, whether a third-party individual intervened on behalf to one of the interacting monkeys (binary: yes vs. no). We tested three predictor variables: social role of signallers (aggressor vs. victim), conflict severity (mild vs. severe) and their interaction. To control for repeated measurements, we included caller identity and context of production (using four levels: natural observations with and without feeding individuals and experiments involving and not involving valuable food items) as random intercepts. After checking for collinearity between variables using correlation matrices (all <0.80), we looked at the normality of residuals and the presence of outliers using graphical analyses of residuals (using half-normal plots and binnedplots).

#### Correcting for multiple testing

Although we ran the analyses on different datasets (all response variables were different), we extracted all acoustic parameters from the same recordings. Since we used a total of 12 linear models, each of them generating one p-value for each of the three fixed effects, we obtained a total of 36 p-values that we adjusted using the false discovery rate (BH method [54]). Although less conservative that the more traditional Bonferroni correction [55], this method is applicable when researchers base their overall decision of the influence of a factor on multiple inferences, as it is the case here since we examined the influence of social role of signallers and conflict severity based on their effect on 12 acoustic features. This method controls both the expected proportion of falsely rejected hypotheses (FDR), and in a weak sense, the more traditional family-wise error rate (FWER), and is thus widely accepted. A commonly used alternative is to first run a PCA to reduce the number of response variables to a smaller set components that are then tested using linear models. However, we did not perform this initial step as we were especially interested in testing predictions derived from animal communication theories [9, 12, 13, 20] concerning the magnitude and direction of effects on specific acoustic parameters. This approach is well established as demonstrated by a number of other studies [56; 57; 58].

All analyses were performed in R version 3.4.2 [47] using RStudio Version 1.1.383 and arm [59], car [60], effects [61], faraway [62], ggpubr [63], lattice [64], lme4 [51], MASS [65], Matrix [66], MuMIN [67] and RVAideMemoire packages [68].

## Results

### Context

Using 187 screams from 13 individuals, we were not able to discriminate screams according to the context of production at the call level. As context, we distinguished whether calls were given during natural agonistic interactions (usually food-related scramble competition) or during artificial feeding events (contest competition; pDFA crossed design: 73.9% expected calls correctly classified compared to 75.5% observed calls correctly classified, *P* = 0.45; cross-validation: 51.5% expected calls correctly cross-classified compared to 56.8% observed calls correctly cross-classified, *P* = 0.15). Similarly, using 102 bouts from 15 individuals, we were not able to discriminate bouts according to the context of production (pDFA crossed design: 59.3% expected calls correctly classified compared to 54.5% observed calls correctly classified, *P* = 0.85; cross-validation 50.5% expected calls correctly cross-classified compared to 41.9% observed calls correctly cross-classified, *P* = 0.88).

### Identity

Following the previous analyses, we pooled the data across contexts for further analyses. Using a fully balanced dataset (social role; conflict severity), we found that screams were individually distinctive at the call level. We found the same pattern even when using an increased sample size (by using an unbalanced crossed design, Table 3). However, we were not able to discriminate screams between individuals at the bout level, including when we increased the sample size (Table 3, detailed results can be found in supplements S7 Appendix).

**Table 3:** Results of DFA investigating individual distinctiveness.

	Call level		Bout level	
	Balanced	Unbalanced	Balanced	Unbalanced
Number of individuals	4	23	8	13
Sample size (screams/bout)	82	296	61	85
Expected calls correctly classified (%)	50.7	34.4	36.1	26.5
Observed calls correctly classified (%)	66.3	50.3	44.1	39.3
*P*	0.02	< 0.001	0.1	0.006
Expected calls correctly cross-classified (%)	24.9	4.4	12.5	7.4
Observed calls correctly cross-classified (%)	47.2	12.9	13.7	12.2
*P* cross-validation	0.002	< 0.001	0.503	0.088

### Social role

**Fig 1:**
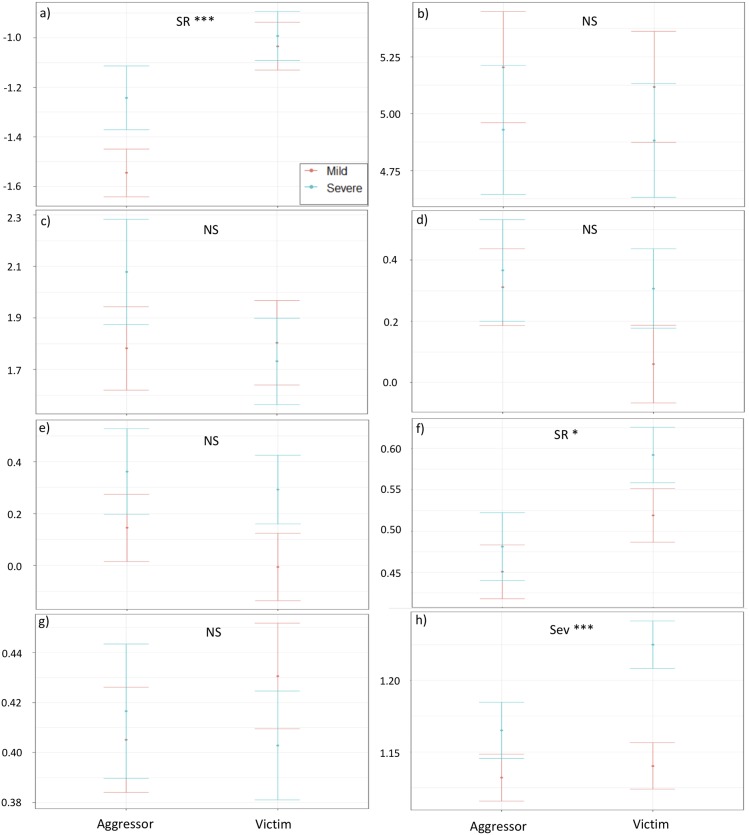
Effect plots displaying results from GLMMs at the call level of eight parameters varying according to the social role of callers (SR: aggressor vs. victim) and conflict severity (Sev: mild in orange vs. severe in blue). a) scream duration (s), b) peak frequency (kHz), c) coefficient of frequency modulation, d) absolute transition onset (Hz), e) absolute transition offset (Hz), f) frequency at quartile 50 (Hz), g) interquartile range (Hz) and h) Shannon entropy. While dots represent the predicted mean of the parameters (previously transformed when necessary and scaled), bars represent standards errors. Significant results are represented by * when adjusted p-values < 0.05, ** <0.01 and *** < 0.001.

**Fig 2:**
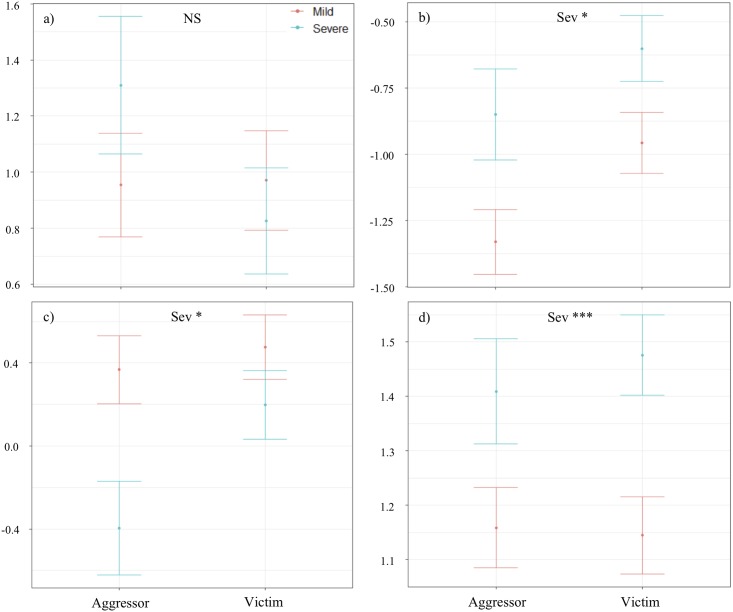
Effect plots displaying results from GLMMs at the bout level of four parameters varying according to the social role of callers (SR: aggressor vs. victim) and conflict severity (Sev: mild in orange vs. severe in blue). a) number of screams, b) average scream duration (s), c) scream rate (number of screams/s) and d) percentage of NLP (%). While dots represent the predicted mean of the parameters (previously transformed when necessary and scaled), bars represent standards errors. Significant results are represented by * when adjusted p-values < 0.05, ** <0.01 and *** < 0.001.

Results from linear models showed that two of eight acoustic parameters tested at the call level were influenced by the social role of signallers (duration and Q50; Table 4 and S8 Appendix). We found that victims produced longer screams and had higher Q50s than aggressors (Fig 1). In line with these results, we found that victims had the tendency to produce longer scream bouts than aggressors (Fig 2, Table 5 and S9 Appendix). However, the percentage of NLP did not differ according to the social role of signallers.

**Table 4:** Results of linear models on the effect of social role and conflict severity at the call level.

Sample size(individuals, screams)	Acoustic features	Social role (binary: aggressor vs. victim)	Severity(binary: mild vs. severe)	Social role: Severity	R^2^m − R^2^c
N = 25N = 301	Duration (s) *Estimate* *SDT valueCI 95%Padjusted p*	0.510.133.910.26 to 0.77 < 0.001< 0.001 ***	0.300.161.94–0.00 to 0.610.1330.318	-0.260.21–1.27–0.66 to 0.140.2030.365	0.07–0.09
N = 25N = 301	Peak frequency (kHz) *Estimate* *SDT valueCI 95%* *P adjusted p*	-0.090.20–0.43–0.48 to 0.310.6520.734	-0.280.25–1.09–0.77 to 0.220.1300.318	0.040.330.12–0.61 to 0.690.9040.904	0.01–0.48
N = 25N = 300	Coefficient of frequency modulation *EstimateSDT valueCI 95%Padjusted p*	0.020.160.14–0.30 to 0.340.3590.538	0.300.211.44–0.11 to 0.700.5480.707	-0.370.27–1.38–0.89 to 0.150.1680.336	0.01–0.60
N = 25N = 301	Absolute transition onset (Hz) *EstimateSDT valueCI 95%Padjusted p*	-0.250.16–1.60–0.56 to 0.060.1480.318	0.050.190.29–0.32 to 0.430.1840.349	0.190.250.77–0.30 to 0.680.4420.636	0.01–0.18
N = 25N = 300	Absolute transition offset (Hz) *EstimateSDT valueCI 95%Padjusted p*	-0.150.15–0.99–0.45 to 0.150.3120.493	0.220.181.19–0.14 to 0.580.0280.112	0.080.240.33–0.40 to 0.560.7390.806	0.02–0.14
N = 24N = 293	Q50 (Hz) *EstimateSDT valueCI 95%Padjusted p*	0.070.041.81–0.01 to 0.140.0030.022 *	0.030.050.67–0.06 to 0.120.0680.222	0.040.060.71–0.08 to 0.160.4780.662	0.05–0.30
N = 25N = 301	IQR (Hz) *Estimate SDT valueCI 95%Padjusted p*	0.030.021.05–0.02 to 0.070.5890.707	0.010.030.39–0.05 to 0.070.5720.707	-0.040.04–1.00–0.12 to 0.040.3150.493	0.005–0.21
N = 25N = 300	Shannon entropy *EstimateSDT valueCI 95%Padjusted p*	0.010.020.50–0.02 to 0.040.022 0.099	0.030.021.67–0.01 to 0.07< 0.001< 0.001 ***	0.050.031.960.00 to 0.100.0500.180	0.10–0.32

Significant results are represented by * when adjusted p-values < 0.05, ** <0.01 and *** < 0.001.

R^2^m corresponds to the variance explained by the fixed effects (marginal); R^2^c corresponds to the variance explained by both the fixed and random effects (conditional). These numbers help to describe the amount of variation explained by the different factors included in the models.

**Table 5:** Results of linear models on the effect of social role and conflict severity at the bout level.

Sample size(individuals, bouts)	Acoustic features	Social role (binary: aggressor vs. victim)	Severity (binary: mild vs. severe)	Social role: Severity	R^2^m − R^2^c
N = 26N = 117	Number of screams *EstimateSDT valueCI 95%Padjusted p*	0.020.210.07–0.40 to 0.440.3120.493	0.360.271.30–0.18 to 0.890.7730.818	-0.500.35–1.44–1.18 to 0.180.1500.318	0.02–0.26
N = 26N = 118	Average scream duration (s)*EstimateSDT value CI 95%Padjusted p*	0.370.162.320.06 to 0.690.0100.051	0.480.202.350.08 to 0.880.0020.018 *	-0.130.26–0.48–0.64 to 0.380.6300.732	0.14–0.19
N = 2N = 118	Scream rate (screams/s) *EstimateSDT valueCI 95%Padjusted p*	0.100.210.52–0.30 to 0.510.0740.222	-0.760.26–2.90–1.28 to -0.250.0050.030 *	0.480.331.45–0.17 to 1.140.1470.318	0.09–0.20
N = 26N = 116	NLP (%) *Estimate SDT valueCI 95%Padjusted p*	-0.010.09–0.16–0.18 to 0.150.8000.823	0.250.112.310.04 to 0.46< 0.001< 0.001 ***	0.080.140.59–0.19 to 0.350.3500.558	0.13–0.34

Significant results are represented by * when adjusted p-values < 0.05, ** <0.01 and *** < 0.001.

R^2^m corresponds to the variance explained by the fixed effects (marginal); R^2^c corresponds to the variance explained by both the fixed and random effects (conditional). These numbers help to describe the amount of variation explained by the different factors included in the models.

### Conflict severity

Results from linear models showed that one of the eight acoustic parameters tested at the call level was influenced by conflict severity (Shannon entropy; Table 4 and S8 Appendix). We found that screams produced during severe aggressions had higher entropy (i.e., were noisier) than the ones produced during mild conflicts (Fig 1). In line with these results, screams produced during severe fights were on average longer in a bout and contained more NLP than the ones produced by individuals facing mild aggressions (Table 5 and S9 Appendix). However, screams were produced at lower rates during severe conflicts (Fig 2).

### Support

**Fig 3:**
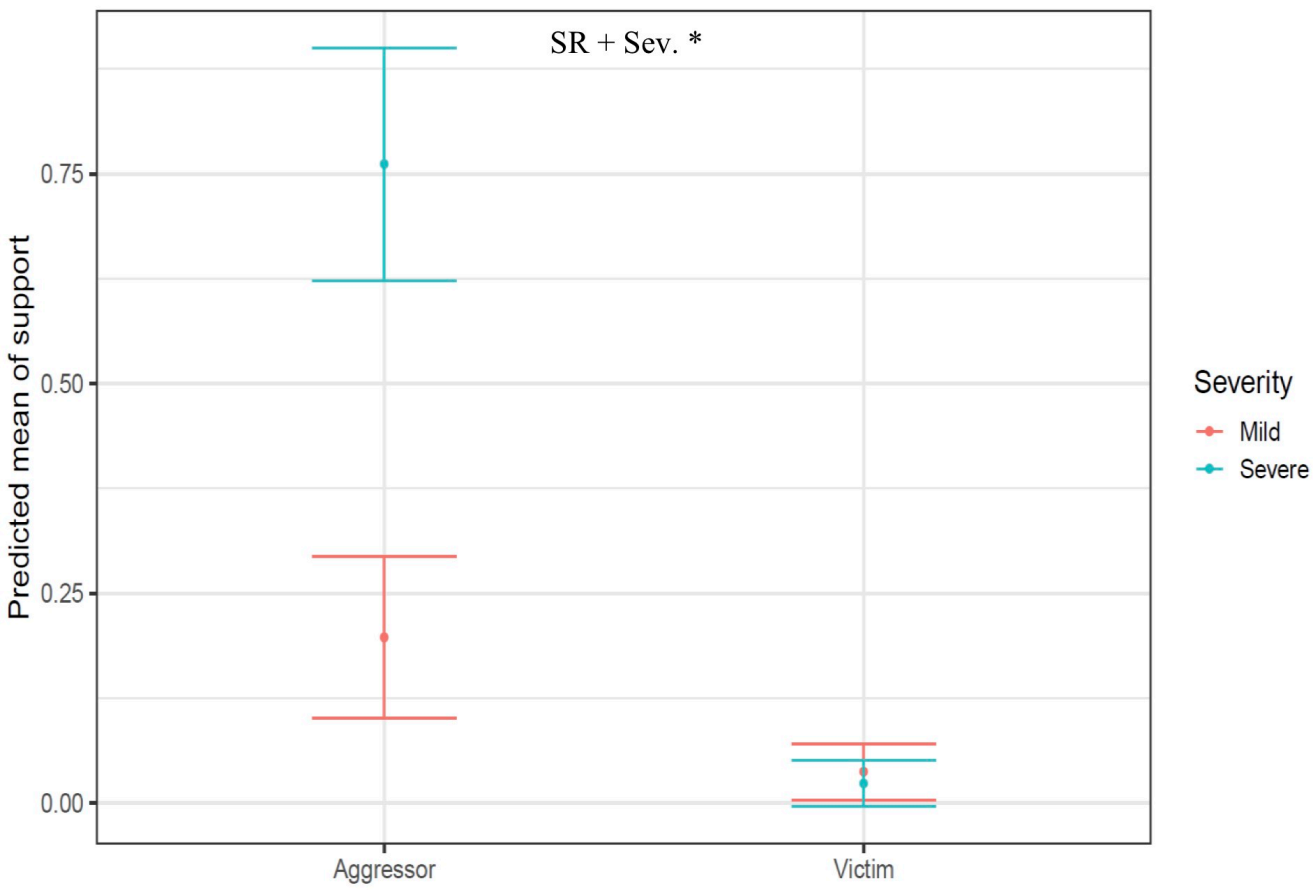
Effect plots displaying results from a GLMM showing the effect of the social role of callers (SR: aggressor vs. victim) and conflict severity (orange: mild aggression, blue: severe aggression) on the occurrence of support at the bout level. While dots represent the predicted mean of the support, bars represent standards errors. Significant results are represented by * when p-values < 0.05.

We used 111 bouts involving 26 callers to investigate the occurrence of support during conflicts according to the social role of signallers (aggressor vs. victim) and conflict severity (mild vs. severe). Supporters intervened in 22 of 111 bouts (19.8%) following scream production. Results from a generalized linear mixed model fitted with a binomial structure and logit-link function showed that both factors influenced the probability of obtaining support (Table 6 and S10 Appendix), with aggressors receiving more support than victims (39.6% vs. 4.8% respectively), and callers being more likely to receive support during severe than mild aggression (27.9% vs. 14.7% respectively, Fig 3).

**Table 6:** Results of a GLMM showing the effect of social role and conflict severity on the occurrence of support offered at the bout level.

Sample size (individuals, bouts)	Amount of support	Social role (binary: aggressor vs. victim)	Severity (binary: mild vs. severe)	Social role: Severity	R^2^m − R^2^c
N = 26N = 111	*EstimateSDZ valueCI 95%P*	-1.860.91–2.05–3.64 to -0.080.040 *	2.561.052.430.49 to 4.620.015 *	-3.041.68–1.81–6.33 to 0.260.071	0.39–0.54

Significant results are represented by * when p-values < 0.05, ** <0.01 and *** < 0.001.

R^2^m corresponds to the variance explained by the fixed effects (marginal); R^2^c corresponds to the variance explained by both the fixed and random effects (conditional). These numbers help to describe the amount of the variation explained by the different factors included in the models.

## Discussion

This study explored how the acoustic structure of wild vervet monkey agonistic screams varied according to identity, social role and conflict severity. We found that calls were individually distinct (at the call but not bout level). We also found that acoustic parameters varied with the caller’s social role and conflict severity. Furthermore, we found that the last two factors also influenced the amount of support signallers received, highlighting the hypothesis that receivers discriminated and responded to these acoustic cues.

The fact that caller identity was encoded at the call but not bout level (S7 Appendix) corroborates previous studies on screams in vervet monkeys [37], Barbary macaques [69] and gorillas, *Gorilla gorilla* [70], suggesting that individual identity is habitually encoded in animal agonistic calls (see [71] for information usually conveyed in primate vocalisations and [72] for a review of vocal individual recognition across species). Scream classification was predominantly based on frequency parameters, i.e., peak frequency, coefficient of frequency modulation and Q50 (S7 Appendix). These results, which mirror those found in other primate species [73, 74], fit within the established pattern describing frequencies and formant dispersion as good indicators of caller identity as they are inversely proportional to the length of the vocal tract of an individual [13, 75, 76]. However, results were not significant at the bout level, that is, we were not able to discriminate individuals at the bout level. Instead, the number of screams and scream rates per bout were more strongly related to the nature of the ongoing event (conflict severity), although consisting of individually identifiable call sequences.

Concerning the social role of callers, we found that screams produced by victims were longer and higher-pitched (higher Q50) than aggressor screams. Furthermore, screams produced during severe conflicts were longer (using average scream duration at the bout level), had higher entropy and a higher percentage of NLP than screams during mild conflicts. This is in agreement with the Morton’s motivational structural rules [9] and Briefer’s emotion hypothesis [20], which both predict that calls produced during hostile situations, i.e., when individuals are aggressors (negative valence), should be of low frequency, having a broadband frequency range (represented here by a high entropy) with a high percentage of NLP. Furthermore, screams produced during high arousal situations, reflected here by conflicts of severe intensity, were expected to be longer. However, in contrast to predictions, screams during severe conflicts were produced at lower rates than screams produced in mild conflicts, when no chasing or physical contact were involved (Tables 4 & 5 and Figs 1 & 2; S8 & S9 Appendices). Both theories predicted them to be produced at higher rates compared to individuals facing less urgent contexts [11], but here we found the opposite pattern, a result also found in wild chimpanzees [44]. One explanation may be that, during severe fights, individuals engage more in physical behaviours, such as being chased/chasing opponents, or fighting behaviours when biting or wrestling [77]. These intense behaviours might affect directly the respiratory system of signallers, and thus reduce their ability to call at high rates.

The acoustic characteristics of vervet monkey screams relate to both the social role of the signallers and to the severity of a conflict, suggesting that they could provide cues for nearby listeners to assess the nature of the ongoing event. Agonistic calls of other primates, such as Geoffroy’s spider monkeys [32] or chimpanzees [34], also differ according to the caller’s social role and conflict severity and playback experiments have confirmed that such cues can be salient to receivers and help bystanders to adapt their responses to intervene only when necessary, that is, to support kin or allies that are involved in severe aggressions [31]. In line with this, the variability of chimpanzee screams can be modulated by the audience. Specifically, call duration and frequency in the second half of the call increased when they were victims of severe attacks, but only when a higher-ranking individual than the opponent was present in the audience. These acoustic variations seemed efficient as callers were then more likely to receive support than individuals that produced non-exaggerated calls [44].

Since we restricted our study to agonistic screams, i.e., produced during agonistic events, other functional hypotheses of primate screams (e.g., to repel predators, advertise copulation or facilitate group fusion) could be excluded [3, 5, 6]. In agonistic contexts, however, screams could function in two mutually non-exclusive ways, i.e., to repel aggressors (e.g. tree shrews [2], several primates [78]) and to recruit allies (e.g. dwarf mongooses [6], pigtail macaques [4], bonnet macaques, *Macaca radiata* [79]). A good illustration of vocalisations fulfilling both functions are chimpanzees calling as victims during conflicts, both to recruit support from bystanders, and to repel their opponent [23].

Our data are in line with Owren & Rendall’s hypothesis [12, 13]. We found that victims produced longer screams that were more piercing (higher Q50) than aggressors. Furthermore, screams produced during severe fights had more noisy components (higher entropy and percentage of NLP) than screams produced in milder conflicts. These characteristics make the calls sound harsher and may help making signallers unappealing targets, ultimately leading to a cessation of negative valence by repelling the aggressor [14]. Specifically, the presence of high percentage of NLP may signal aggressive motivation [18] and directly affect listener physiology [12], leading to avoidance [13, 14]. Simultaneously, the harshness of screams due to NLP can also prevent habituation [80, 81], signal the physical condition of signallers [18] and facilitate individual recognition [19]. At the same time, these characteristics could thus also help to increase the attentional state of third-party listeners [13] and provide crucial cues necessary to increase signallers’ chances of obtaining support, a dual function.

Although further playback experiments are needed to test the dual-function hypothesis, research on screams produced by juvenile vervet monkeys in Amboseli National Park in Kenya suggests that these calls are efficient in recruiting support, as mothers approached and threatened opponents in 22% of conflicts involving their screaming offspring [37]. In our study, we also found that bystanders provided support in 19.8% of the interactions involving screams. Importantly, our results showed that both the social role of signaller and conflict severity influenced the probability of receiving support, with aggressors receiving more supports than victims, and callers being more likely to receive support during severe fights (S10 Appendix). While we cannot exclude the possibility that nearby listeners visually assessed the interaction, in some cases future supporters were unable to see the conflict, suggesting that they relied on the calls’ acoustic characteristics when taking a decision as to whether or not to intervene.

Although victims would benefit more from bystander than aggressors (due to higher risks of injuries), we found that aggressors received more support than victims (39.6% vs 4.8% respectively). This is in line with other evidence showing that vervet monkeys primarily support the higher-ranking individual in a conflict, which is usually the aggressor [38]. Furthermore, from the helper’s perspective, it might be costlier to intervene in favour of victims than aggressors, potentially explaining why aggressors generally receive more support than victims. However, it is important to point out that during conflicts vervet monkeys frequently switch social roles: individuals screaming for help may start as victims and, as soon as they receive aid, effectively become aggressors by chasing away their opponents due to the coalitionary support they received.

Vervet monkeys tend to form coalitions with kin or allies and when outranking their opponents [82]. Since aggressors were more likely to receive support than victims, it is unlikely that victims screamed to recruit help but rather to repel opponents [13–15]. Alternatively, victim screams could be part of a multimodal display to signal submission, in a similar way that canids can use yelp in situations of high intensity submission [83]. In vervet monkeys, victims often crouch or expose their teeth when facing their opponents, suggesting they are mainly motivated to indicate subordination to reduce further aggression [73, 84–86]. In doing so, victims might avoid further escalation, and further support for aggressors. Vervet monkey screams, in other words, are likely to serve different functions, depending on the social role of the signallers.

## Conclusions

We demonstrated that variation in acoustic parameters is related to the caller’s identity, social role and conflict severity. We have also demonstrated that vervet monkey screams may serve a dual function during conflicts, both as signals aimed at the opponent and at bystanders. While non-linearities and noisy components in screams appear to create aversive responses in opponents, variation in duration and frequency-related parameters appear to be directed at nearby listeners to solicit their help. Beyond these effects, it would be interesting to examine the impact of further social factors on the acoustic structure, such as the age and social rank of the callers [87], the social relationships in dyads [8] or the presence of specific social partners as bystanders [44]. Such social and other environmental factors are likely to have further impact, which will help to a better understanding of the functions and mechanisms underlying socially targeted vocal behaviour.

## Supporting information

S1 Appendix Vervet monkey screams. (DOCX)

S2 Appendix Detailed predictions for each acoustic parameter used in this study. (DOCX)

S3 Appendix Description of call providers. (DOCX)

S4 Appendix Acoustic data. (DOCX)

S5 Appendix Ethogram of the behaviours used to define the social roles of signaller. (DOCX)

S6 Appendix Inter-Observer reliability test. (DOCX)

S7 Appendix Detailed results of DFA on caller identity. (DOCX)

S8 Appendix Detailed results of analyses at the call level. (DOCX)

S9 Appendix Detailed results of analyses at the bout level. (DOCX)

S10 Appendix Detailed results of the support analysis. (DOCX)
