# Supplementary material for: Correlates of social role and conflict severity in wild vervet monkey agonistic screams
Source: PLoS One. 2019 May 1;14(5):e0214640. doi: 10.1371/journal.pone.0214640 (PMC6493722; doi:10.1371/journal.pone.0214640)
Supplement: S1 Appendix — (DOCX) [file pone.0214640.s001.docx]

## S1. Vervet monkey screams

In order to study the influence of social role of signallers and conflict severity on the acoustic structure of vocal signals produced by wild vervet monkeys, we focused on all high-pitched calls produced during agonistic encounters, categorised by Struhsaker as squeals, chutter-squeals, squeal-screams and screams (Table S1, 1967). However, for more clarity, we grouped all those signals occurring during agonistic encounters under “screams” and we did not take into account all other scream-like vocalisations produced by this species in other contexts in our study.

**Table S1. Description of different types of screams produced by vervet monkeys**

| Context | Call types | Acoustic features | Main callers | Bystanders reaction |
| --- | --- | --- | --- | --- |
| Agonistic encounters | Squeals | Uniform, tonal calls | Adult females and juveniles | Support caller |
|  | Chutter-squeals | Mixed phases with tonal and non-tonal calls |  |  |
|  | Squeal-screams | Non-tonal sounds superimposed by tonal band |  |  |
|  | Screams | Non-tonal shrill-sounding calls |  |  |
| Mating | Anti-copulatory squeal-screams | Resemble squeal-screams | Adult and sub-adult females | Move away from females, copulation inhibited |
| Approach of a “strange” male | Screams | High-pitched shrill-sounding calls | Infants | Approach and retrieval from mothers, males moving away |
| Weaning | Weaning squeals | Compound units, ultrasonic portions in calls | Infants | Mothers refusing nursing, moving away from infants |
|  | Weaning screams | Non-tonal calls |  |  |
| Separation from the rest of group | Lost squeals | Mix of tonal and non-tonal portions intermingled with lost rrr and lost Rrah | Infants | No obvious reaction apart from mothers sometimes facilitating reunion by locating and retrieving their infants |
|  | Lost screams | Similar to weaning screams and intermingled with lost squeals and lost rrr |  |  |

modified from Struhsaker (1967)
