# Supplementary material for: Correlates of social role and conflict severity in wild vervet monkey agonistic screams
Source: PLoS One. 2019 May 1;14(5):e0214640. doi: 10.1371/journal.pone.0214640 (PMC6493722; doi:10.1371/journal.pone.0214640)
Supplement: S2 Appendix — (DOCX) [file pone.0214640.s002.docx]

## S2. Detailed predictions

**Table S2. Predictions of the influence of the social role of signallers (aggressor vs. victim) and conflict severity (mild vs. severe) on 15 selected acoustic parameters**

| Parameters | Predictions | References |
| --- | --- | --- |
| CALL LEVEL | | |
| Scream duration (s) | Longer call in urgent context:  victim > aggressor  severe > mild | - Slocombe and Zuberbuhler (2005, 2007); Taylor and Reby (2010) |
| Peak frequency (kHz) | Lower peak frequency sound when hostile and low-urgency context:  victim > aggressor  severe > mild | Morton (1977); Slocombe and Zuberbuhler (2005, 2007); Taylor and Reby (2010); Walter and Schnitzler (2017) |
| Coefficient of frequency variation * | Rapid fluctuation evokes attention:  victim > aggressor  severe > mild | Morton (1977); Tooze, Harrington, and Fentress (1990); Owren and Rendall (2001); Briefer (2012); E. C. Déaux, Clarke, and Charrier (2016) |
| Coefficient of frequency modulation | Rapid fluctuation evokes attention:  victim > aggressor  severe > mild | Morton (1977); Tooze et al. (1990); Owren and Rendall (2001); Briefer (2012); E. C. Déaux et al. (2016) |
| Absolute transition onset (Hz) | Sound rising in frequency indicates lower hostility:  victim > aggressor  severe > mild | Morton (1977); Slocombe and Zuberbuhler (2005, 2007) |
| Absolute transition offset (Hz) | Sound decreasing in frequency indicates higher hostility:  victim < aggressor  severe > mild | Morton (1977); Slocombe and Zuberbuhler (2005, 2007) |
| Frequency quartile 50 (Hz) | Higher frequency quartile sound when submissive:  victim > aggressor  severe > mild | Morton (1977); Taylor and Reby (2010); É. C. Déaux, Charrier, and Clarke (2016); Walter and Schnitzler (2017) |
| Inter-quartile range (Hz) | Tonal sound (represented by a lower inter-quartile range) when fearful:  victim < aggressor  severe > mild | Morton (1977); Briefer (2012); É. C. Déaux et al. (2016); Walter and Schnitzler (2017) |
| Shannon entropy | Noisy call (high-entropy) produces aversive response:  victim > aggressor  severe > mild | Morton (1977); S. Gouzoules et al. (1984); Ordóñez-Gómez et al. (2015) |
| BOUT LEVEL | | |
| Bout duration (s) * | Longer in urgent context:  victim > aggressor  severe > mild | Morton (1977); Slocombe and Zuberbuhler (2007); Taylor and Reby (2010) |
| Number of screams | Higher in high intensity situation:  victim > aggressor  severe > mild | Rendall (2003); Bastian and Schmidt (2008); Taylor and Reby (2010); Clay, Ravaux, de Waal, and Zuberbühler (2016) |
| Average scream duration (s) | Longer in urgent context:  victim > aggressor  severe > mild | Morton (1977); Slocombe and Zuberbuhler (2005, 2007); Taylor and Reby (2010) |
| Scream intervals (s) * | Shorter in urgent context:  victim > aggressor  severe > mild | Morton (1977); Rendall (2003); Bastian and Schmidt (2008); Taylor and Reby (2010); Briefer (2012) |
| Scream rate (number of screams/s) | Higher in high arousal situation:  victim > aggressor  severe > mild | Seyfarth and Cheney (2003a, 2003b); Taylor and Reby (2010); Briefer (2012) |
| Percentage of screams with NLP (%) | Higher in high arousal situation:  victim > aggressor  severe > mild | Riede et al. (2007); Blumstein and Recapet (2009); Townsend and Manser (2011); Clay et al. (2016) |

* Parameters excluded due to high correlations or failure to reach symmetrical distribution, leading to analyses using 12 acoustic parameters (eight at the call level and four at the bout level).
