# Supplementary material for: Correlates of social role and conflict severity in wild vervet monkey agonistic screams
Source: PLoS One. 2019 May 1;14(5):e0214640. doi: 10.1371/journal.pone.0214640 (PMC6493722; doi:10.1371/journal.pone.0214640)
Supplement: S3 Appendix — (DOCX) [file pone.0214640.s003.docx]

## S3. Call providers

**Table S3. Description of call providers, number of events, bouts and screams recorded for each individual and their participation in our study, i.e., whether we recorded their screams in both social role (aggressor vs. victim) and conflict severity (mild vs. severe), as well as whether we used them in call and/or bout analyses**

| Caller | Age | Sex | Number of events | Number of bouts | Number of screams | Both social role  (aggressor vs. victim) | Both severity  (mild vs. severe) | Used for call analyses | Used for bout analyses |
| --- | --- | --- | --- | --- | --- | --- | --- | --- | --- |
| Asis | Adult | Female | 2 (6) | 3 (7) | 5 (10) | Yes | No | X | √ |
| Enge | Adult | Female | 8 | 9 | 15 (16) | Yes | Yes | √ | √ |
| Heer | Adult | Female | 1 | 2 | 2 | Yes | No | √ | √ |
| Miel | Adult | Female | 4 | 4 | 17 (4) | Yes | Yes | √ | √ |
| Mooi | Adult | Female | 5 (7) | 5 (7) | 17 (22) | Yes | Yes | √ | √ |
| Ouli | Adult | Female | 6 | 9 | 10 (12) | Yes | Yes | √ | √ |
| Pann | Adult | Female | 3 | 3 | 8 (2) | Yes | Yes | √ | √ |
| Riss | Adult | Female | 3 | 3 | 9 (2) | Yes | Yes | √ | √ |
| Aapi | Juvenile | Female | 2 (3) | 2 (4) | 17 (34) | Yes | Yes | √ | √ |
| Alsi | Juvenile | Female | 3 | 3 | 8 (5) | Yes | Yes | √ | √ |
| Hipp | Juvenile | Female | 6 | 8 | 16 (17) | Yes | Yes | √ | √ |
| LBlind | Juvenile | Female | 2 (3) | 5 (6) | 18 (27) | Yes | Yes | √ | √ |
| Nies | Juvenile | Female | 8 | 8 | 15 (12) | Yes | Yes | √ | √ |
| Piep | Juvenile | Female | 6 | 9 | 19 (10) | Yes | Yes | √ | √ |
| Potj | Juvenile | Female | 2 | 2 (3) | 21 (27) | Yes | Yes | √ | √ |
| Afr | Juvenile | Male | 5 (7) | 8 (10) | 19 (42) | Yes | Yes | √ | √ |
| Bul | Juvenile | Male | 3 | 3 | 6 (1) | No | Yes | √ | √ |
| Nok | Juvenile | Male | 3 | 3 | 6 (4) | Yes | Yes | √ | √ |
| Ogi | Juvenile | Male | 5 (6) | 6 (7) | 26 (48) | Yes | Yes | √ | √ |
| Onb | Juvenile | Male | 3 | 5 | 25 (7) | Yes | Yes | √ | √ |
| Poe | Juvenile | Male | 2 (4) | 2 (4) | 15 (22) | Yes | Yes | √ | √ |
| Roo | Juvenile | Male | 4 | 5 | 32 (26) | Yes | Yes | √ | √ |
| Spo | Juvenile | Male | 4 (7) | 5 (8) | 21 (48) | Yes | Yes | √ | √ |
| Vak | Juvenile | Male | 3 (4) | 3 (4) | 13 (27) | Yes | Yes | √ | √ |
| Wol | Juvenile | Male | 1 | 2 | 5 (6) | Yes | No | √ | √ |
| Wur | Juvenile | Male | 1 | 2 | 9 (6) | Yes | No | √ | √ |
|  |  |  | *95 (47)* | *119*  *(60)* | *374*  *(437)* | *N = 25 individuals* | *N = 22 individuals* | *N = 25 individuals* | *N = 26 individuals* |

Numbers in brackets represent the number of data that had been excluded from the analyses due to poor signal to noise ratio. A tick mark (√) in the last two columns indicates that calls produced by an individual have been used for the concerned analyses, while a cross mark (X) indicates that we had to exclude calls from an individual due to bad quality or lack of samples.
