# Supplementary material for: Correlates of social role and conflict severity in wild vervet monkey agonistic screams
Source: PLoS One. 2019 May 1;14(5):e0214640. doi: 10.1371/journal.pone.0214640 (PMC6493722; doi:10.1371/journal.pone.0214640)
Supplement: S4 Appendix — (DOCX) [file pone.0214640.s004.docx]

## S4. Acoustic data

## Sound inspection in Praat

After selecting raw recordings of good quality from a maximum of individuals in as many situations as possible, i.e., recorded in both social roles (aggressor vs. victim) and conflict severity (mild vs. severe), we inspected sounds and created corresponding text grids (Table S4) in Praat 5.4.13 (Boersma 2006, www.praat.org), using a Fast Fourier Transformation (Hanning window shape, window length = 0.01s, number of time steps = 1000, number of frequency steps = 500 and dynamic range = 40dB). When possible, we also selected screams that opposed the same two individuals during the same period (or with minimal time intervals) in different contexts in order to obtain a balanced dataset minimizing potential differences due to external factors, such as identities of opponents or recording day. Comparisons of text grids created by two different observers (Fig S1 and S2) allowed us to test for inter-observer reliability on acoustic data using Cohen’s method, making sure that data were extracted in a reliable way (1960; see S6).

**Table S4. Description of the main steps used to annotate recordings, which allowed us to extract events, bouts and calls, for which precise acoustic parameters could be measured**

| Main steps | Praat commands |
| --- | --- |
| Open original recording (wav file) | Open - Read from file… |
| Select sound and create the appropriate TextGrid | Annotate – To TextGrid… |
| Define all tiers names | Remplace “Mary John bell” by tiers used for the study, i.e. here “Context Event Bouts Calls Type NLP PreSuffix Breaks”, leaving blank the second question concerning the point tiers |
| Edit the TextGrid | Select both the sound and the corresponding TextGrid and click on “View & Edit” |
| Annotate Tiers | - **Context**: define whether the vocalisations were recorded during natural observations (OB) or during feeding experiments (Exp), adding more details when possible on the precise context, i.e., the behaviour of the caller or the kind of experiment involved - **Event**: delimit starting and ending point in order to obtain the duration of an event (EventDuration) - **Bouts**: delimit starting and ending point of each bout, allowing us to get the number of bouts and their duration (NbBout and BoutDuration), and noting down the event type for each bout, i.e., whether the signaller was aggressor or victim and whether the conflict was of mild or severe aggression. - **Calls**: delimit starting and ending point of each vocal unit, separated by other ones by at least 0.03s of silence, and noting the call type produced when known such as screams, aggressive calls, grunts or NA if not clear. In order to keep only calls of good quality for the analyses, we also noted here whether the calls were of bad quality (BadSc), clipped (ClippedSc), if some other individuals were calling simultaneously during a chorus (ChorSc), or if there was a lot of background noise such as birds (Sc-B), insects (Sc-I) or humans (Sc-H). Consequently, only screams with clear annotations (sc) were extracted and used in further analyses. - **Type**: define the social role of the signaller and conflict severity for each call, using the four following categories: aggressor of mild conflict (AscM), agressor of severe aggression (AscS), victim of mild conflict (VscM) and victim of severe fight (VscS). - **NLP**: define whether some irregularities occur within each call, indicating when possible the kind of NLP encountered: frequency jumps (FJ), sub-harmonics (SH), deterministic chaos (DC), periodic window (PW) and bi-phonation (BP; Fig S2) - **PreSuffix**: define whether a prefix and/or suffix was observed at the beginning and/or end of each call respectively - **Breaks**: define whether a break (B), i.e. a silence of less than 0.03s, occurred within each call |
| Save the TextGrid | Save TextGrid as text file, making sure to keep exactly the same name for the TextGrid and the corresponding sound file |


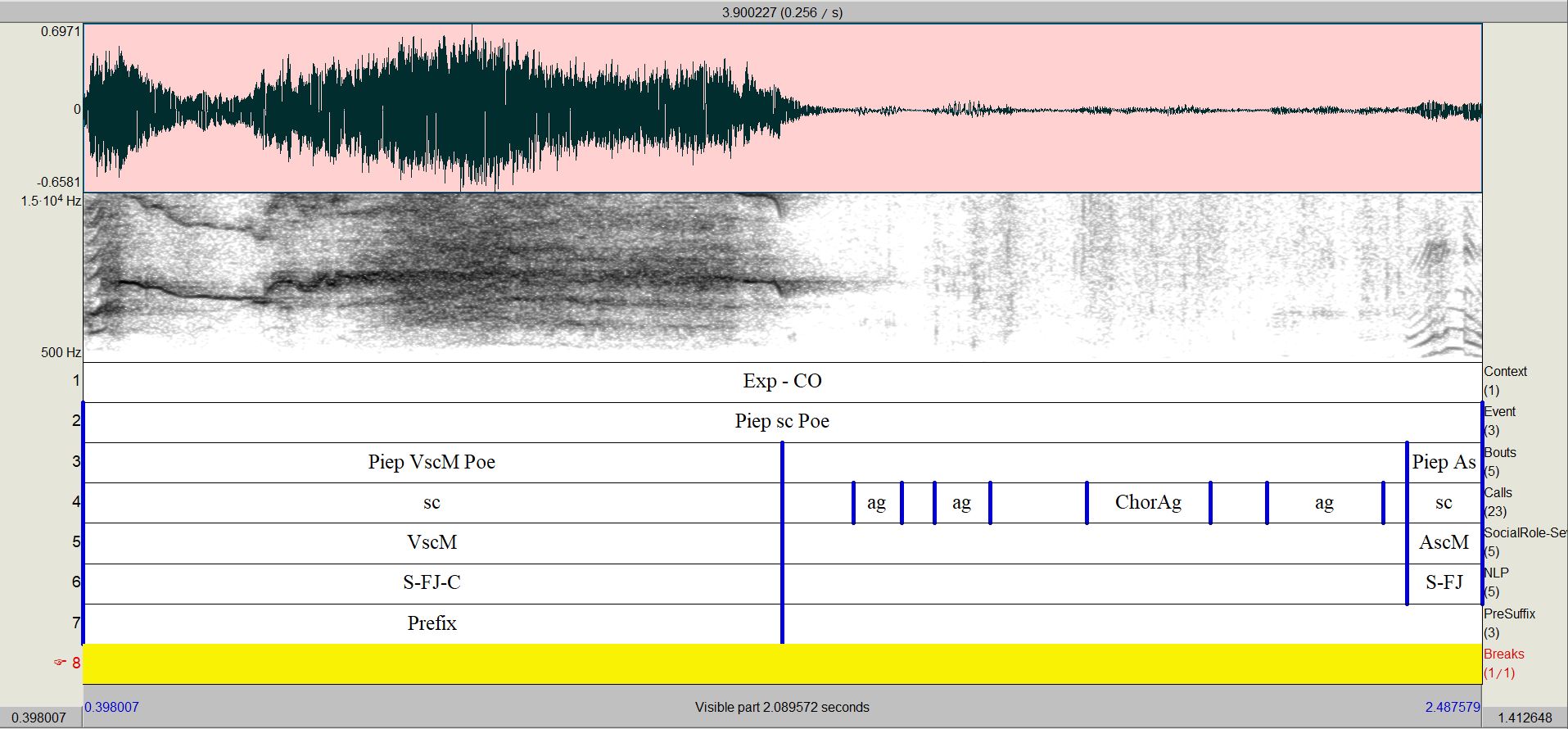


**Fig S1. Example of a raw recording recorded around an experiment with its corresponding annotated text grid** (in order to make the last scream more visible, we adapted here the settings in Praat to 60dB). While a victim of a mild conflict screamed at the beginning of the event (left side), the social role changed, and the signaller became an aggressor of a mild conflict who again screamed at the end of the event (right side). As this social role switching defined a new bout, we thus analysed here one event containing two bouts and two screams (one scream in each bout). Note: we also defined new bouts, not only when the social role switched, but also when the severity of the conflict changed.


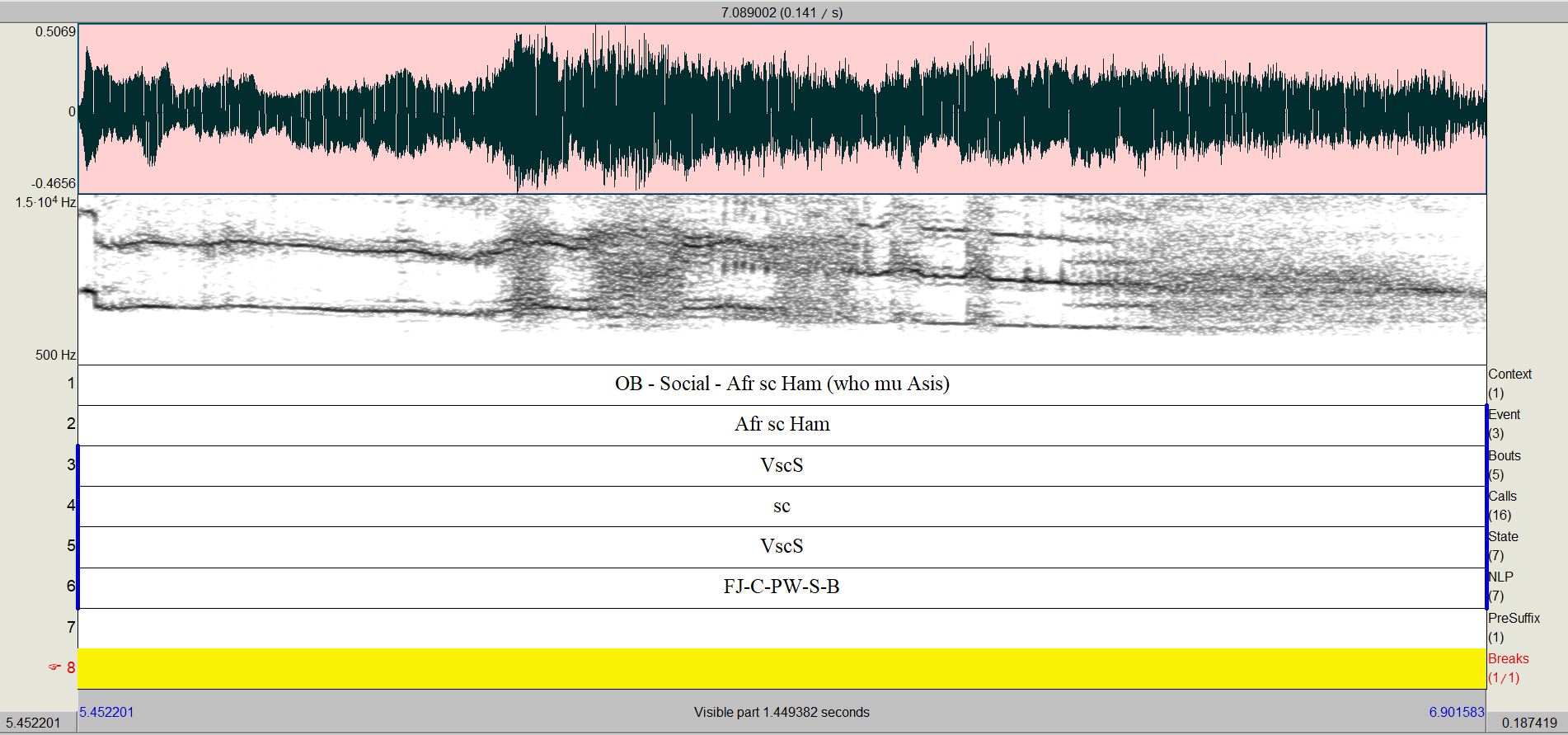


1 2 3 2 3 4 3 4 3 2/3 4 2 3 2 4 2 5 4

**Fig S2. Example of a spectrogram and its corresponding annotated text grid showing non-linear phenomena found in a scream produced by a juvenile male vervet monkey when being victim of a severe conflict**. Different numbers at the bottom of the figure represent the different classes of NLP: 1) frequency jumps, 2) periodic window, 3) sub-harmonics, 4) deterministic chaos and 5) bi-phonation.

## Acoustic parameters extraction in R

As we were unable to use fundamental and formant frequencies due to the noisy acoustic components often present in vervet monkey screams, we selected 15 acoustic parameters that described the acoustic properties of their screams and the temporal structure of their bouts (nine parameters at the call and six at the bout level; Table 2 and S2). While temporal parameters were extracted from spectrograms and oscillograms created in Praat 5.4.13 (Boersma 2006, www.praat.org) using a Fast Fourier Transformation (Hanning window shape, window length = 0.01s, number of time steps = 1000, number of frequency steps = 500 and dynamic range = 40dB), all other acoustic parameters were extracted from spectrograms and spectral slices (Fig S3) with the following settings: sampling rate of 44.1 kHz, 16 bits accuracy, Fast Fourier Transformation with 512 samples, Hanning window and 90% overlap using Seewave (Sueur, Aubin et al. 2008) and tuneR packages (Ligges 2013) in R version 1.0.143 (Team 2016).

| 1. Aggressor of a mild conflict   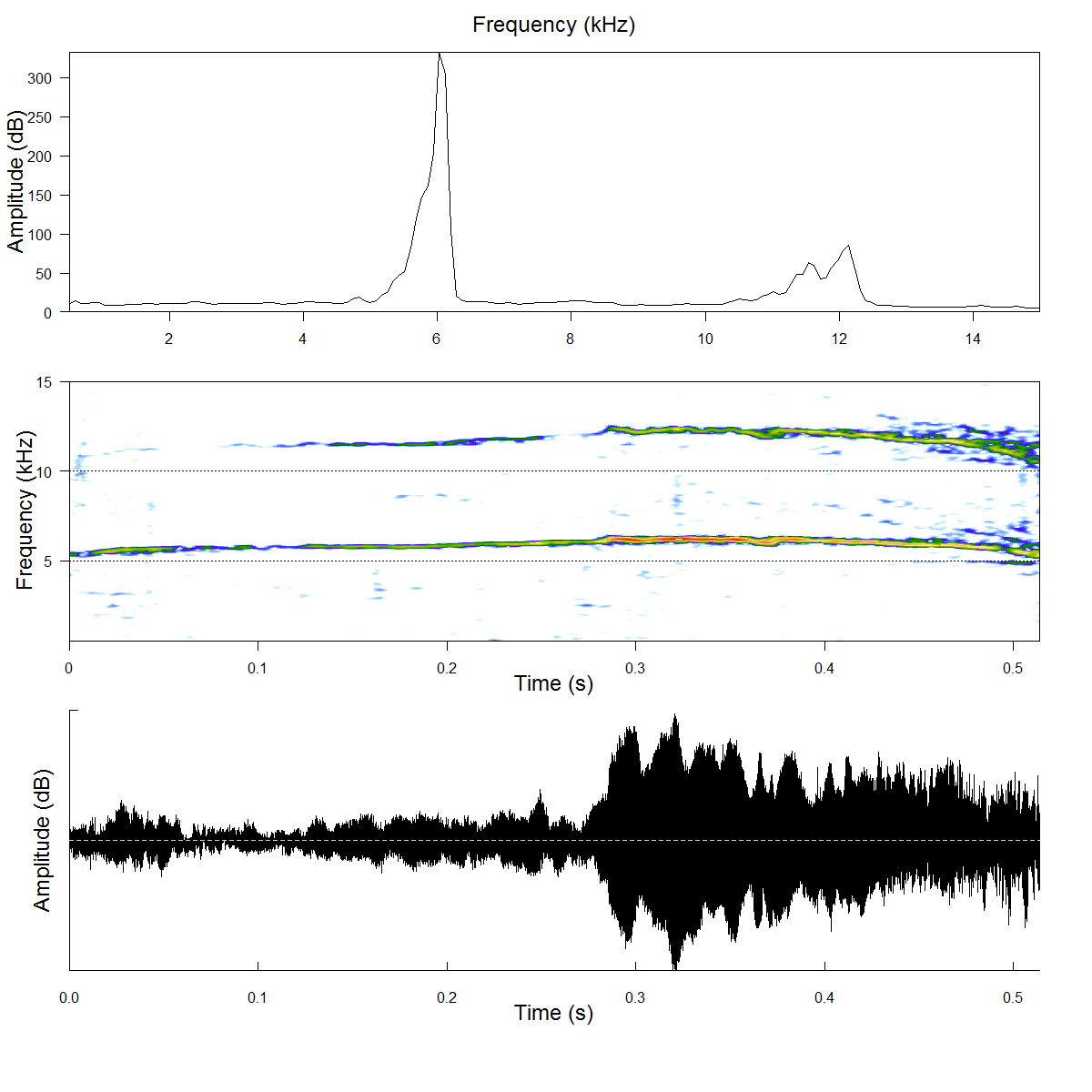 | 1. Aggressor of a severe conflict   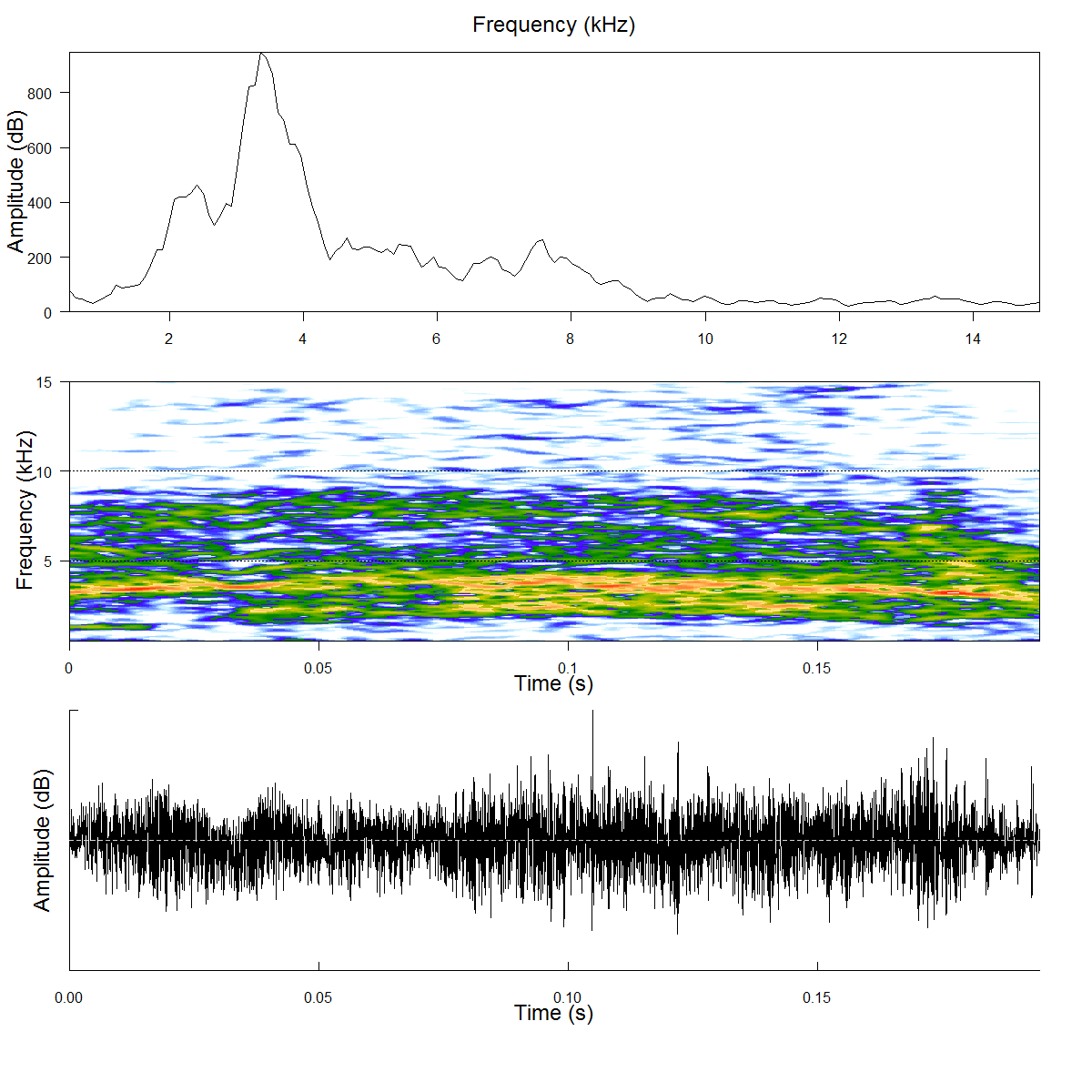 |
| --- | --- |
| 1. Victim of a mild conflict   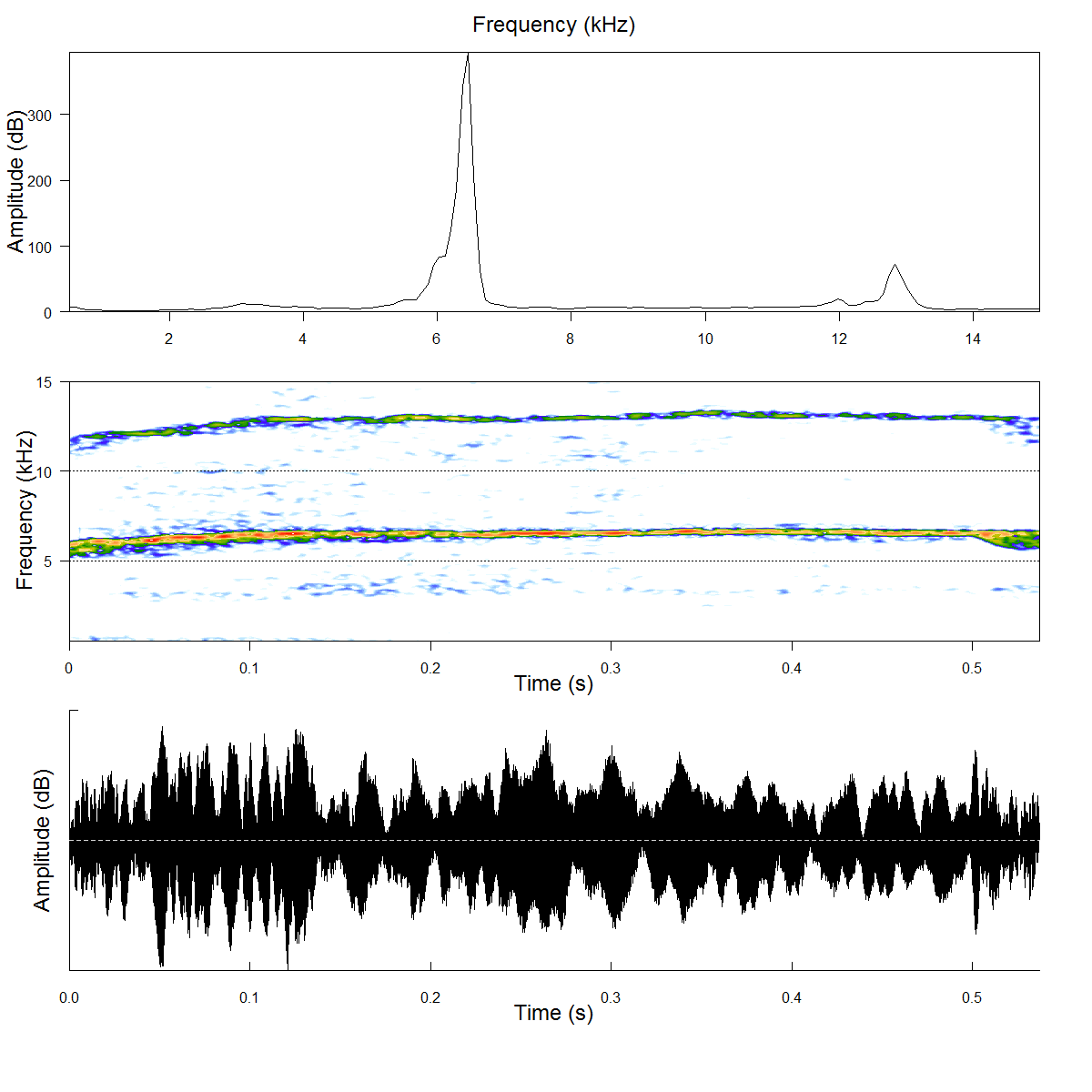 | 1. Victim of a severe conflict   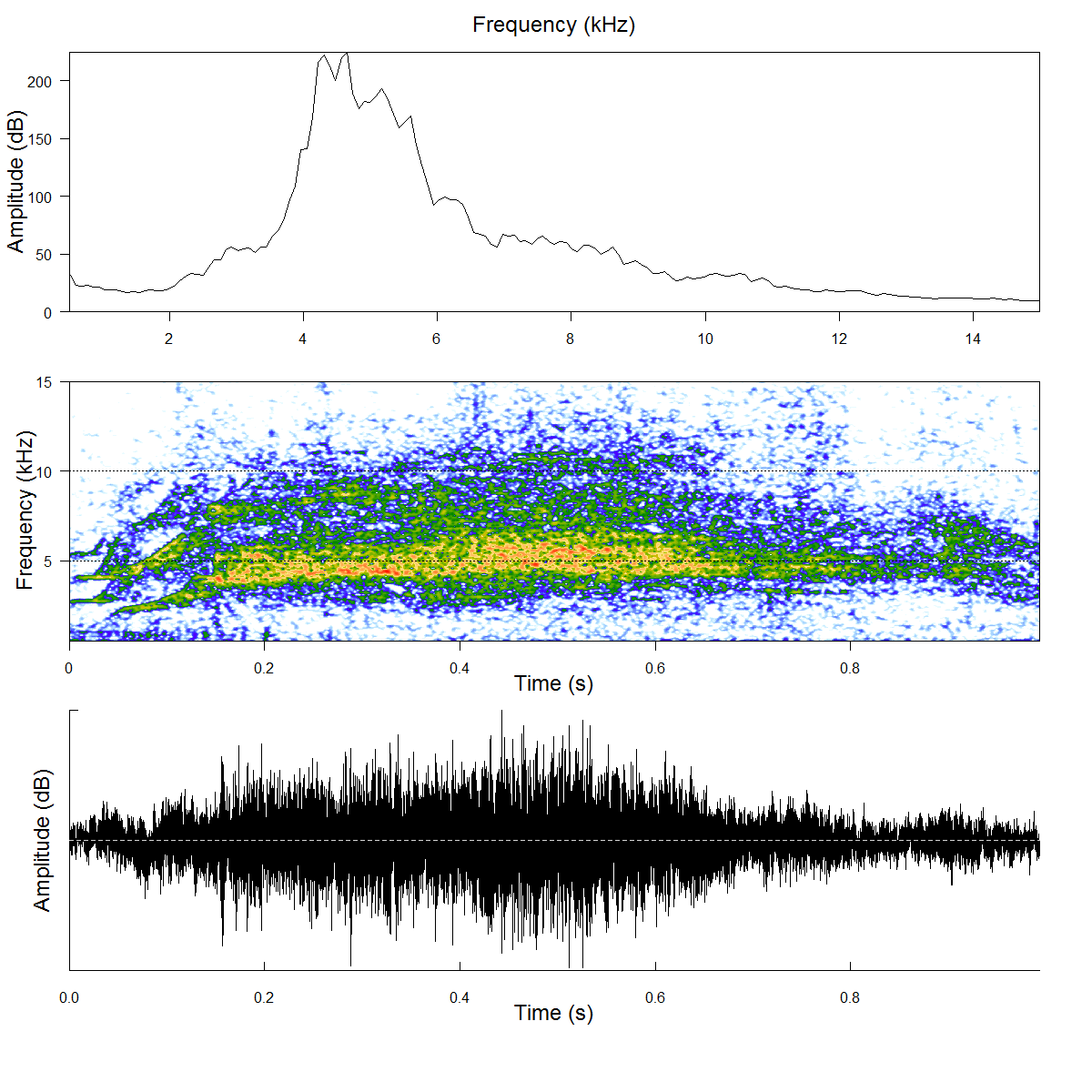 |

**Fig S3. Example of spectrograms, oscillograms and spectral slices obtained using R of screams produced by different individuals in all event types (social role and conflict severity).**
