# Supplementary material for: Correlates of social role and conflict severity in wild vervet monkey agonistic screams
Source: PLoS One. 2019 May 1;14(5):e0214640. doi: 10.1371/journal.pone.0214640 (PMC6493722; doi:10.1371/journal.pone.0214640)
Supplement: S5 Appendix — (DOCX) [file pone.0214640.s005.docx]

## S5. Detailed ethogram

**Table S5. Ethogram of the behaviours used to define the social role of signallers**

| **Social Role** | **Behaviour** | **Code** | **Description** |
| --- | --- | --- | --- |
| Aggressor | Aggressive calls | ag | Producing aggressive vocalisations |
|  | Attack | at | Forward motion of the body towards the opponent, often combined with stare (st.at) |
|  | Bite | bi | Biting another individual, often combined with grab (gb.bi) |
|  | Chase | ch | Running after another individual who is fleeing |
|  | Displace | tp | Individual takes the place of another who retreats, usually combined with approach (ap.tp) |
|  | Grab | gb | Grabbing another individual with the hand and/or the mouth |
|  | Hit | hi | Hitting another individual |
|  | Monopolise | mo | Keeping a valuable item such as food away from other interested individuals |
|  | Stare | st | Popping up the eyelids, exposing the white above the eyes, often combined with attack (st.at) |
| Victim | Avoid | av | Stopping previous behaviour or moving the head or body away from an aggressor |
|  | Crawl | cr | Crouching down while facing an aggressor |
|  | Flee | fl | Running away from an aggressor that is chasing |
|  | Frustration hop | fh | Jumping on the spot while looking at an individual usually monopolising a valuable resource, often while screaming and combined with standing up |
|  | Jump aside | ja | Jumping aside from the opponent |
|  | Leave | le | Walking away from an aggressor |
|  | Look for support | lo | Looking around for support while being threatened by an aggressor |
|  | Redirect | re | Performing some aggressive behaviours after being a victim, targeting a third uninvolved individual that is becoming the new victim |
|  | Retreat | rt | Quickly leaving the proximity of another individual, without running and/or moving large distances |
|  | Stand up | su | Standing up during a conflict, often following a crawl and combined with frustration hop and screams |
