# Supplementary material for: Correlates of social role and conflict severity in wild vervet monkey agonistic screams
Source: PLoS One. 2019 May 1;14(5):e0214640. doi: 10.1371/journal.pone.0214640 (PMC6493722; doi:10.1371/journal.pone.0214640)
Supplement: S6 Appendix — (DOCX) [file pone.0214640.s006.docx]

S6. Inter-observer reliability tests
a) Behavioural data

For each event included in a row (S6 Table), two observers (SM & EW) specified independently for each bout (ranging from one to three), the social role of signallers (aggressor vs. victim) as well as the conflict severity (mild vs. severe) when clear (NA if we could not determine it precisely) using one of the following categories:

- A = scream produced by an aggressor of an aggression
- V = scream produced by a victim of an aggression
- M = scream produced during a mild aggression
- S = scream produced during a severe aggression

**Table S6. Example of dataset to be filled independently to calculate Cohen’s Kappa**

| Behavioural description | BOUT 1 | | BOUT 2 | | BOUT 3 | | BOUT 4 | | NbBouts |
| --- | --- | --- | --- | --- | --- | --- | --- | --- | --- |
|  | SR1 | Sev1 | SR2 | Sev2 | SR3 | Sev3 | SR4 | Sev4 |  |
| Afr voc.sc Heer rt |  |  |  |  |  |  |  |  |  |
| Afr st.sc.vo Tor rt |  |  |  |  |  |  |  |  |  |
| distr call seq afrikans against |  |  |  |  |  |  |  |  |  |
| Nok aps c.rt Ogi st.at |  |  |  |  |  |  |  |  |  |
| Miel st.gb.bi Nies ja.sc |  |  |  |  |  |  |  |  |  |

We then compared the data sheets from the two observers in order to observe the percentage of agreement between both researchers, thus making sure that we collected data in the same way, suggesting that they were meaningful for analyses. We calculated Cohen’s Kappa (Cohen 1960) by comparing the percentage of correct agreement observed with the one expected by chance for the number of bouts defined by each researcher for each event (Fig S4), as well as for the social role of signallers (Fig S5) and the conflict severity (Fig S6) for each bout. We used an average of 50.7% of the data to calculate Cohen’s Kappa score and accepted data as correctly collected if the scores reached a minimum of 80% agreement between the two observers.

**Fig S4. Picture of datasheet prepared to calculate the Cohen’s Kappa for the number of bouts observed in events**. We calculated proportion of agreement observed as the sum of each diagonal cells corresponding to correct agreement (i.e. yellow cells) divided by the sum of each total row (i.e. Totals- Row 1+2+3+4), while we calculated proportion of agreement expected by chance as the sum of the multiplication Totals-Column*Totals-Row (i.e Row-Column Product) divided by the multiplication of Row-Totals + Column-Totals.

**Fig S5. Picture of datasheet prepared to calculate the Cohen’s Kappa for the social role of signallers for each bout observed in events**. We calculated proportion of agreement observed as the sum of each diagonal cells corresponding to correct agreement (i.e. yellow cells) divided by the sum of each total row (i.e. Totals- Row V+A+NA), while we calculated proportion of agreement expected by chance as the sum of the multiplication Totals-Column*Totals-Row (i.e Row-Column Product) divided by the multiplication of Row-Totals + Column-Totals.

**Fig S6. Picture of datasheet prepared to calculate the Cohen’s Kappa for conflict severity for each bout observed in events**. We calculated proportion of agreement observed as the sum of each diagonal cells corresponding to correct agreement (i.e. yellow cells) divided by the sum of each total row (i.e. Totals- Row M+S+NA), while we calculated proportion of agreement expected by chance as the sum of the multiplication Totals-Column*Totals-Row (i.e Row-Column Product) divided by the multiplication of Row-Totals + Column-Totals.

b) Acoustic data

For each recording, two observers (SM & ED) annotated corresponding text grids independently in Praat in order to define precisely the starting and ending points of each “Event”, “Bouts” (ranging from one to four) and “Calls” (ranging from one to over 20, see Fig S1. in S4 Appendix for an example of annotated TextGrid of a recording). Each observer had then to complete an excel table (S7 Table) in order to obtain some relevant measurements, i.e. the duration of the event (Event duration), the total number of screams produced in the event (Nb screams), the mean duration of screams in an event (Sc duration), the number of screams of good quality that can be used for further analyses (Analysable screams, excluding screams annotated as bad quality or for which there was a lot of background noise), and the percentage of screams including NLP within the event (NLP% corresponding to (number of screams with NLP/total number of screams within an event)*100). Please note that as we usually had a single event per recording, researchers in the field starting a new recording after each conflict, we did not assess inter-observer reliability on this parameter.

**Table S7. Example of dataset to be filled independently to calculate Cohen’s Kappa**

| Wav File | Event duration (s) | Nb screams | Sc duration (s) | Analysable screams | NLP (%) |
| --- | --- | --- | --- | --- | --- |
| Afr_VscM_Neu_17nov14 |  |  |  |  |  |
| Afr_AscM_Tor_5jul14 |  |  |  |  |  |
| Afr_sc_Unk_17jul12 |  |  |  |  |  |
| Afr_Asc_Ham_7Aug14 |  |  |  |  |  |
| Afr_Vsc_Che_18-juil-14 |  |  |  |  |  |

The comparison of those data sheets filled up by the two observers allowed us to observe directly whether some bias due to an observer could be made while describing agonistic interactions, using the percentage of agreement between both researchers. To make sure that we collected the data in similar way, suggesting that they were meaningful for analyses, we calculated Cohen’s Kappa (Cohen, 1960) by comparing the percentage of correct agreement observed with the one expected by chance for each measure, as for example here shown for the duration of the event (Fig S7). Although we accepted an interval of one second to define the duration of the event, we had to agree within 0.03s for screams’ durations as by definition, a silence of at least 0.03s allowed us to distinguish two separated screams. We used 14% of the data to calculate Cohen’s Kappa score and accepted data as correctly collected if the scores reached a minimum of 70% agreement between the two observers.

**Fig S7. Picture of datasheet prepared to calculate the Cohen’s Kappa for the duration of event obtained from annotated text grids of recordings**. We calculated proportion of agreement observed as the sum of each diagonal cells corresponding to correct agreement (i.e. yellow cells) divided by the sum of each total row (i.e. Totals- Row), while we calculated proportion of agreement expected by chance as the sum of the multiplication Totals-Column*Totals-Row (i.e. Row-Column Product) divided by the multiplication of ROW-TOTALS + COLUMN-TOTALS.
