# Supplementary material for: Correlates of social role and conflict severity in wild vervet monkey agonistic screams
Source: PLoS One. 2019 May 1;14(5):e0214640. doi: 10.1371/journal.pone.0214640 (PMC6493722; doi:10.1371/journal.pone.0214640)
Supplement: S7 Appendix — (DOCX) [file pone.0214640.s007.docx]

## S7. Results from DFAs on caller identity

1. DFA on caller identity at the call level – conservative approach

(using a balanced dataset with N = 4 individuals & N = 82 screams)

| Prior probabilities of groups   \| Afr \| Ogi \| Onb \| Piep \| \| --- \| --- \| --- \| --- \| \| 0.171 \| 0.293 \| 0.305 \| 0.232 \| | Proportion of trace   \| LD1 \| LD2 \| \| LD3 \| \| --- \| --- \| --- \| --- \| \| 0.508 \| 0.290 \| 0.203 \| \| |
| --- | --- | --- | --- | --- | --- | --- | --- | --- | --- | --- | --- | --- | --- | --- | --- | --- | --- |

Group means

| Callers | Duration | PF | CoFm | Onset | Offset | Q50 | IQR | Sh |
| --- | --- | --- | --- | --- | --- | --- | --- | --- |
| Afr | 0.007 | -0.063 | -0.563 | -0.440 | -0.791 | -0.157 | -0.109 | -0.117 |
| Ogi | -0.245 | 0.397 | -0.270 | 0.180 | 0.007 | -0.108 | -0.383 | -0.260 |
| Onb | -0.080 | -0.574 | 0.520 | -0.203 | 0.243 | -0.222 | -0.043 | 0.005 |
| Piep | 0.411 | 0.301 | 0.072 | 0.364 | 0.255 | 0.544 | 0.621 | 0.409 |

Coefficients of linear discriminants

| Parameters | LD1 | LD2 | LD3 |
| --- | --- | --- | --- |
| Duration | -0.262 | 0.052 | 0.648 |
| PF | -0.800 | -0.207 | -0.320 |
| CoFm | 0.979 | 0.113 | -0.301 |
| Onset | -0.198 | -0.270 | -0.225 |
| Offset | 0.348 | -0.654 | -0.443 |
| Q50 | 0.680 | -0.783 | -0.353 |
| IQR | -0.241 | -0.495 | 0.718 |
| Sh | -0.013 | 0.478 | 0.392 |


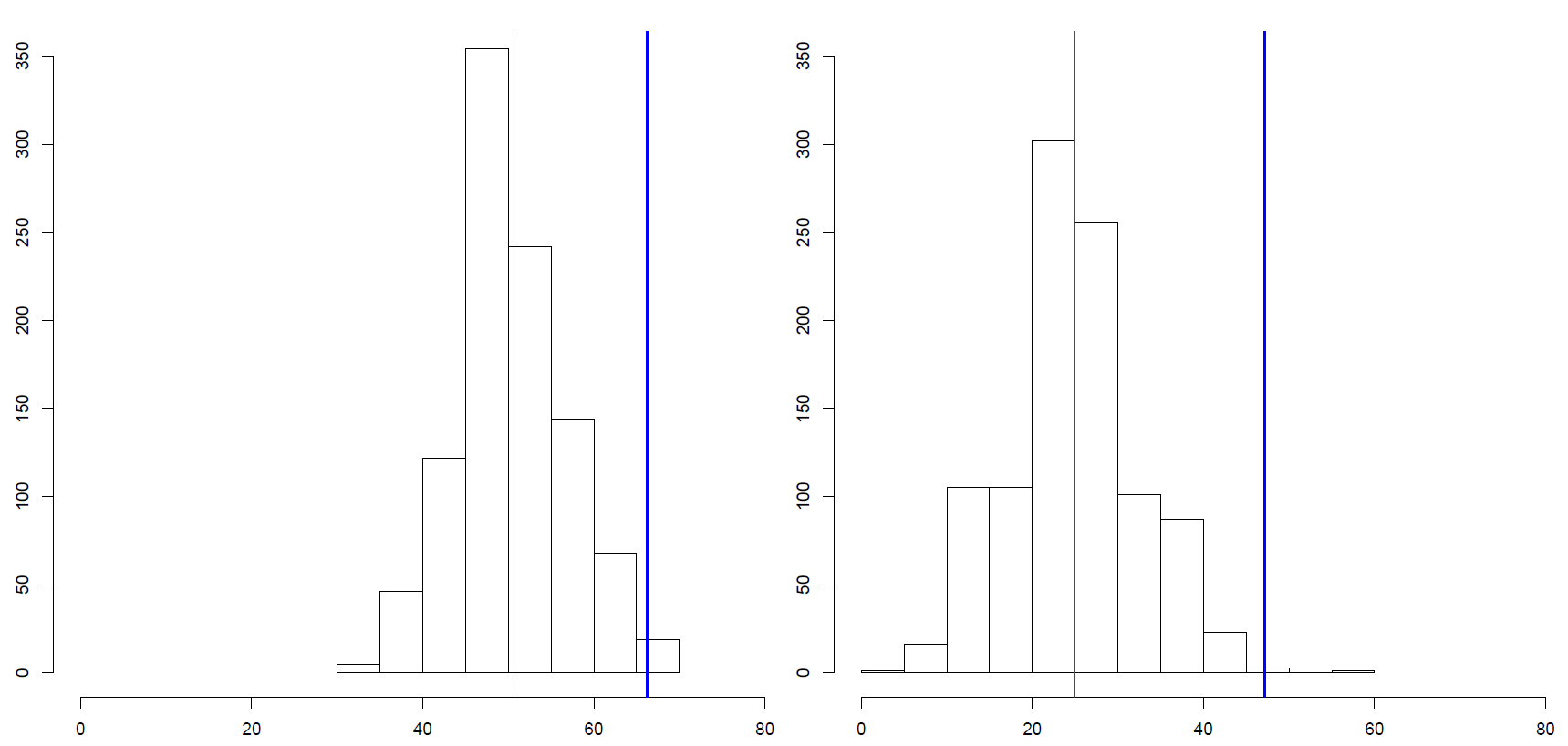


b)

a)

**Fig S8. Histograms showing the frequency of classification rates from DFAs (%) at the call level with a balanced dataset using a) correct classification and b) correct cross-classification.** While red bars correspond to the expected percentage of correctly classified, respectively cross-classified calls, blue bars represent the mean percentage of correct classified and cross-classified calls obtained from real dataset.

**
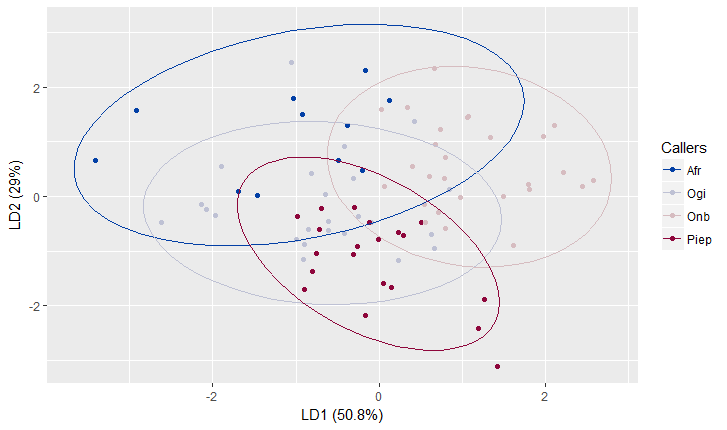
Fig S9. Graph showing ﬁrst and second discriminant component scores resulting from an analysis of 82 screams produced by four individuals, represented by the different colours.** Both axes represent the percentage of the variance explained by the ﬁrst and second discriminant functions, respectively.

1. DFA on caller identity at the call level – incomplete crossed design
   (using an incomplete crossed design with N = 21 individuals & N = 301 screams)

Prior probabilities of groups

| Aapi | Afr | Alsi | Bul | Enge | Hipp | Hwa | LBlind | Miel | Mooi | Nies | Nok |
| --- | --- | --- | --- | --- | --- | --- | --- | --- | --- | --- | --- |
| 0.030 | 0.047 | 0.027 | 0.020 | 0.050 | 0.043 | 0.017 | 0.056 | 0.056 | 0.037 | 0.050 | 0.020 |
| Ogi | Onb | Ouli | Pann | Piep | Poe | Riss | Roo | Spo | Vak | Wol | Wur |
| 0.080 | 0.083 | 0.033 | 0.027 | 0.063 | 0.017 | 0.030 | 0.106 | 0.027 | 0.037 | 0.017 | 0.030 |

Group means

| Callers | Duration | PF | CoFm | Onset | Offset | Q50 | IQR | Sh |
| --- | --- | --- | --- | --- | --- | --- | --- | --- |
| Aapi | -0.167 | 0.522 | 0.097 | -0.479 | -0.471 | 0.681 | 1.082 | 0.671 |
| Afr | -0.072 | 0.170 | -0.465 | -0.254 | -0.575 | -0.081 | 0.146 | 0.013 |
| Alsi | 0.406 | 0.569 | -0.506 | 0.293 | -0.459 | 1.178 | 0.322 | 0.839 |
| Bul | 0.499 | 0.785 | -1.299 | -0.142 | -0.351 | 0.567 | -0.306 | 0.153 |
| Enge | 0.084 | -0.959 | 0.122 | -0.429 | -0.181 | -0.217 | -0.382 | 0.404 |
| Hipp | -0.370 | 0.359 | -0.123 | 0.262 | 0.072 | 0.253 | 0.025 | 0.080 |
| Hwa | -0.126 | -0.587 | -0.803 | -0.689 | -0.375 | -0.148 | -0.646 | 0.140 |
| LBlind | -0.005 | 0.945 | 0.690 | 0.477 | 0.380 | -0.108 | 0.368 | -0.519 |
| Miel | -0.084 | -0.083 | 0.269 | 0.465 | 0.588 | 0.052 | 0.062 | 0.352 |
| Mooi | 0.258 | -1.094 | -0.330 | -0.183 | -0.511 | -0.476 | -0.118 | -0.151 |
| Nies | -0.044 | -0.207 | -0.264 | -0.025 | 0.205 | 0.167 | -0.537 | 0.318 |
| Nok | 0.204 | -0.615 | -0.299 | -0.917 | -0.997 | -0.492 | -0.275 | -0.168 |
| Ogi | -0.299 | 0.654 | -0.183 | 0.389 | 0.197 | -0.029 | -0.140 | -0.133 |
| Onb | -0.151 | -0.366 | 0.580 | -0.008 | 0.426 | -0.151 | 0.214 | 0.138 |
| Ouli | -0.179 | -0.367 | -0.594 | 0.094 | -0.030 | -0.060 | -0.483 | -0.424 |
| Pann | -0.267 | -0.058 | -0.259 | 0.234 | 0.593 | 0.159 | -0.341 | 0.465 |
| Piep | 0.291 | 0.553 | 0.147 | 0.580 | 0.438 | 0.669 | 0.905 | 0.553 |
| Poe | 0.787 | 0.766 | 0.074 | -0.315 | 0.472 | 0.529 | 0.703 | 0.703 |
| Riss | -0.331 | -0.925 | 0.470 | -0.120 | -0.654 | -0.652 | -0.562 | -0.268 |
| Roo | 0.226 | -0.634 | 0.701 | -0.041 | 0.065 | -0.572 | 0.179 | -0.428 |
| Spo | -0.568 | -0.883 | 0.373 | -0.533 | 0.105 | -0.452 | -0.271 | -0.070 |
| Vak | 0.963 | 0.525 | -0.061 | -0.048 | -0.182 | -0.142 | -0.444 | -0.258 |
| Wol | -0.267 | -0.395 | -1.894 | -1.126 | -0.516 | -0.081 | -0.599 | -1.276 |
| Wur | -0.779 | 1.061 | -0.631 | -0.462 | -1.001 | 0.578 | -0.376 | -0.747 |

Coefficients of linear discriminants

| Parameters | LD1 | LD2 | LD3 | LD4 | LD5 | LD6 | LD7 | LD8 |
| --- | --- | --- | --- | --- | --- | --- | --- | --- |
| Duration | 0.290 | -0.105 | -0.139 | 0.893 | 0.407 | 0.174 | 0.027 | -0.428 |
| PF | -1.134 | 0.374 | -0.311 | 0.253 | 0.380 | -0.661 | 0.257 | 0.251 |
| CoFm | 0.385 | 0.742 | 0.380 | -0.191 | -0.325 | -0.631 | -0.032 | -0.541 |
| Onset | -0.058 | 0.100 | 0.338 | 0.119 | 0.202 | 0.333 | -1.031 | 0.196 |
| Offset | -0.126 | 0.332 | -0.001 | -0.484 | 0.488 | 0.554 | 0.684 | -0.098 |
| Q50 | -0.193 | -0.476 | 0.696 | -0.764 | -0.831 | 0.495 | -0.150 | -1.222 |
| IQR | -0.196 | 0.219 | 0.021 | 0.538 | -0.570 | 0.663 | 0.126 | 0.531 |
| Sh | 0.289 | 0.587 | -1.400 | 0.014 | 0.276 | -0.592 | -0.081 | 0.474 |

Proportion of trace

| LD1 | LD2 | | LD3 | LD4 | LD5 | LD6 | LD7 | LD8 |
| --- | --- | --- | --- | --- | --- | --- | --- | --- |
| 0.389 | 0.216 | 0.112 | | 0.092 | 0.073 | 0.060 | 0.033 | 0.025 |

b)

a)

**
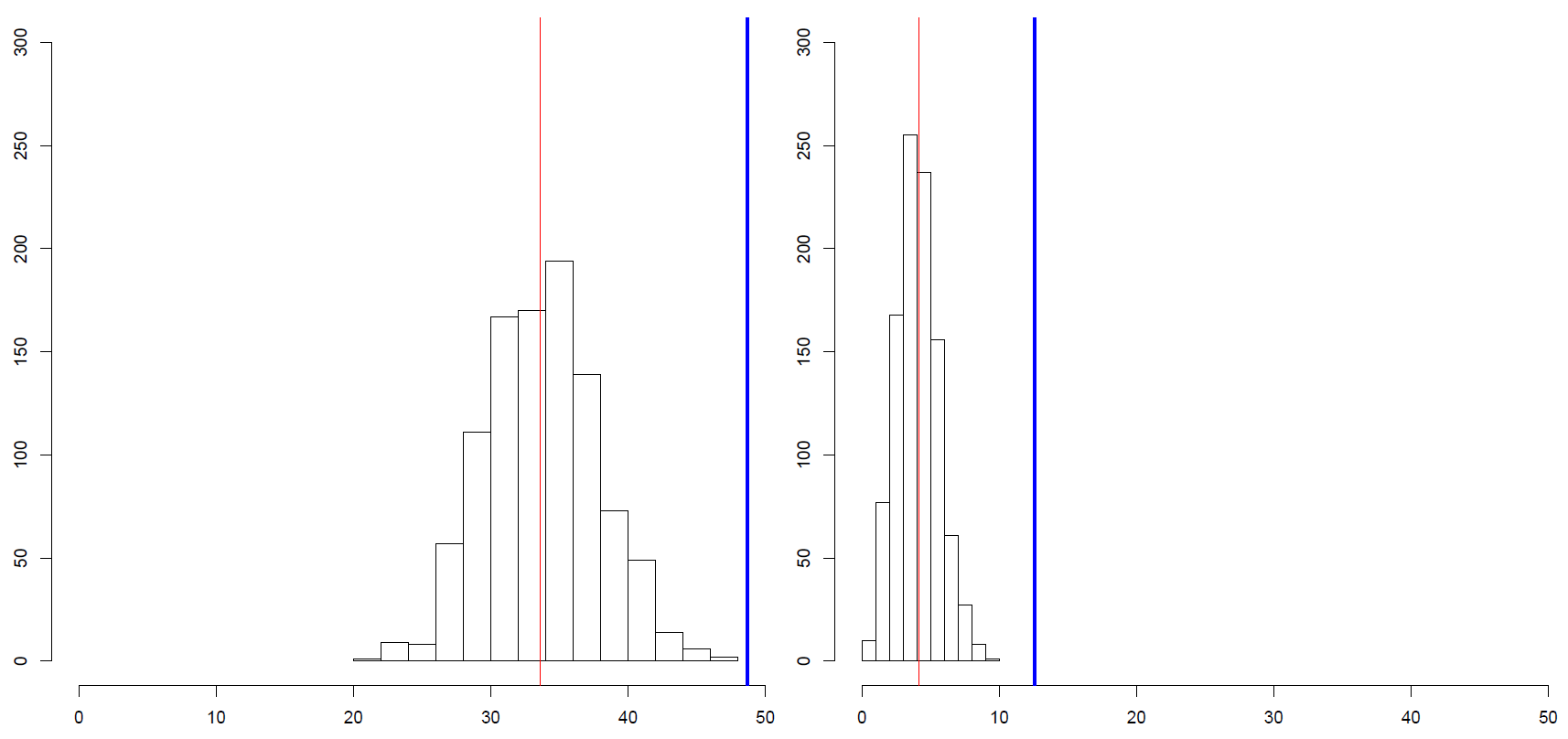
Fig S10. Histograms showing the frequency of classification rates from DFAs (%) at the call level with an incomplete crossed-design using a) correct classification and b) correct cross-classification.** While red bars correspond to the expected percentage of correctly classified, respectively cross-classified calls, blue bars represent the mean percentage of correct classified and cross-classified calls obtained from real dataset.

**
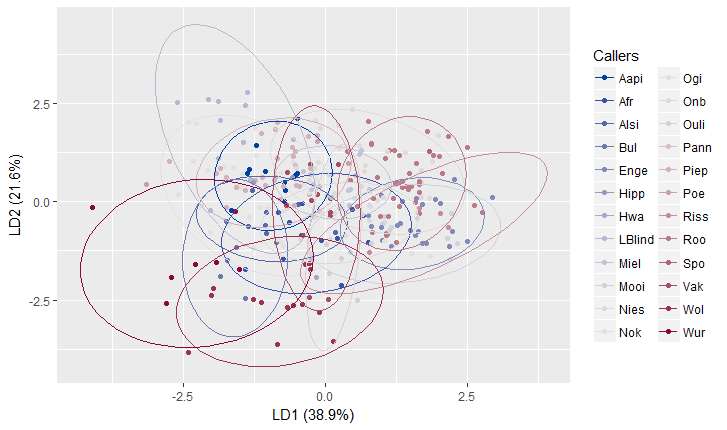
Fig S11. Graph showing ﬁrst and second discriminant component scores resulting from an analysis of 301 screams produced by 21 individuals, represented by the different colours.** Both axes represent the percentage of the variance explained by the ﬁrst and second discriminant functions, respectively.

- 1. DFA on caller identity at the bout level – conservative approach
     (using a balanced dataset with N = 8 individuals & N = 61 bouts)

Prior probabilities of groups

| Afr | Enge | Hipp | Nies | Ogi | Onb | Ouli | Piep |
| --- | --- | --- | --- | --- | --- | --- | --- |
| 0.131 | 0.148 | 0.131 | 0.131 | 0.098 | 0.082 | 0.131 | 0.148 |

Group means

| Callers | NbSc | ScDuration | ScRate | NLP |
| --- | --- | --- | --- | --- |
| Afr | -0.155 | 0.472 | -0.577 | 0.437 |
| Enge | 0.006 | -0.165 | 0.492 | 0.504 |
| Hipp | 0.357 | -0.470 | 0.057 | -0.029 |
| Nies | -0.445 | 0.386 | 0.055 | -0.010 |
| Ogi | 0.593 | -0.293 | -0.431 | 0.009 |
| Onb | 0.677 | 0.023 | -0.273 | 0.161 |
| Ouli | -0.365 | -0.478 | 0.668 | -0.984 |
| Piep | -0.237 | 0.427 | -0.234 | -0.079 |

Coefficients of linear discriminants

| Parameters | LD1 | LD2 | LD3 | LD4 |
| --- | --- | --- | --- | --- |
| NbSc | 0.389 | 0.894 | 0.360 | 0.917 |
| ScDuration | -0.060 | -0.227 | 0.989 | 1.056 |
| ScRate | -0.517 | 0.742 | 1.217 | 0.574 |
| NLP | 0.910 | 0.144 | 0.389 | -0.444 |

Proportion of trace

| LD1 | LD2 | | LD3 | LD4 |
| --- | --- | --- | --- | --- |
| 0.506 | 0.285 | 0.174 | | 0.036 |

b)

a)

**
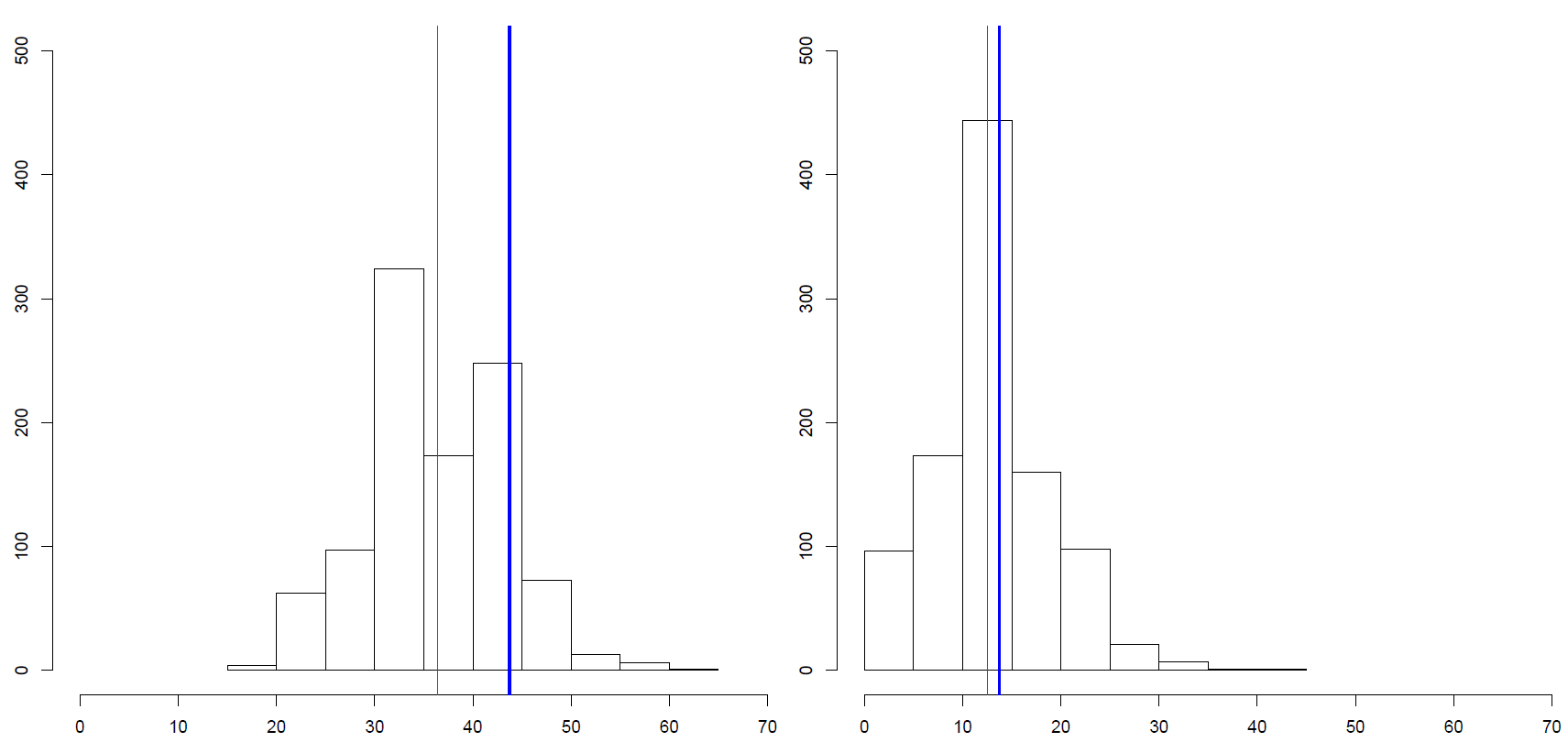
Fig S12. Histograms showing the frequency of classification rates from DFAs (%) at the bout level with a balanced dataset using a) correct classification and b) correct cross-classification.** While red bars correspond to the expected percentage of correctly classified, respectively cross-classified calls, blue bars represent the mean percentage of correct classified and cross-classified calls obtained from real dataset.


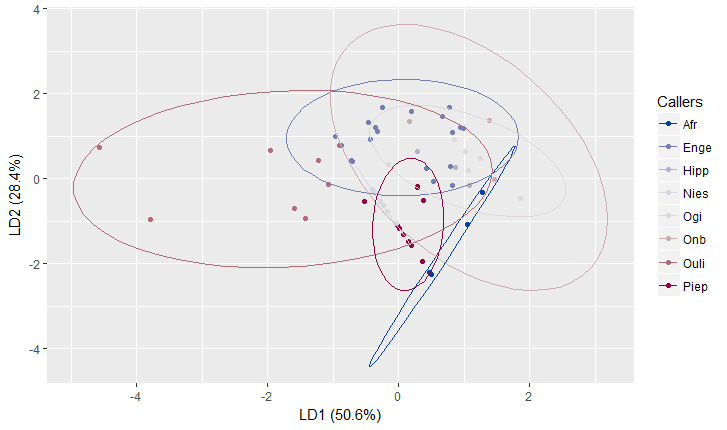


**Fig S13. Graph showing ﬁrst and second discriminant component scores resulting from an analysis of 61 bouts produced by eight individuals, represented by the different colours.** Both axes represent the percentage of the variance explained by the ﬁrst and second discriminant functions, respectively.

1. DFA on caller identity at the bout level – incomplete crossed design
   (using an incomplete crossed design with N = 13 individuals & N = 85 bouts)

Prior probabilities of groups

| Afr | Enge | Hipp | LBlind | Miel | Mooi | Nies | Ogi | Onb | Ouli | Piep | Roo | Spo |
| --- | --- | --- | --- | --- | --- | --- | --- | --- | --- | --- | --- | --- |
| 0.094 | 0.106 | 0.094 | 0.059 | 0.047 | 0.059 | 0.094 | 0.071 | 0.059 | 0.094 | 0.106 | 0.059 | 0.059 |

Group means

|  | NbSc | ScDuration | ScRate | NLP |
| --- | --- | --- | --- | --- |
| Afr | -0.313 | 0.481 | -0.688 | 0.530 |
| Enge | -0.146 | -0.150 | 0.492 | 0.599 |
| Hipp | 0.216 | -0.452 | 0.012 | 0.052 |
| LBlind | 0.008 | 0.083 | 0.331 | 0.404 |
| Miel | 0.602 | -0.287 | 0.010 | -0.114 |
| Mooi | -0.588 | 0.552 | -0.370 | 0.364 |
| Nies | -0.613 | 0.397 | 0.010 | 0.072 |
| Ogi | 0.461 | -0.276 | -0.527 | 0.091 |
| Onb | 0.547 | 0.037 | -0.352 | 0.247 |
| Ouli | -0.529 | -0.460 | 0.686 | -0.928 |
| Piep | -0.397 | 0.437 | -0.310 | 0.000 |
| Roo | 1.403 | 0.019 | -0.213 | -0.260 |
| Spo | 0.159 | -1.476 | 0.878 | 0.091 |

Coefficients of linear discriminants

| Parameters | LD1 | LD2 | LD3 | LD4 |
| --- | --- | --- | --- | --- |
| NbSc | 1.053 | 0.464 | 0.614 | 0.035 |
| ScDuration | -0.046 | 0.284 | 1.373 | 0.462 |
| ScRate | 0.718 | -0.410 | 0.885 | 0.893 |
| NLP | 0.163 | 0.779 | -0.571 | 0.614 |

Proportion of trace

| LD1 | LD2 | | LD3 | LD4 |
| --- | --- | --- | --- | --- |
| 0.379 | 0.311 | 0.193 | | 0.117 |

b)

a)

**Fig S14. Histograms showing the frequency of classification rates from DFAs (%) at the bout level with an incomplete crossed-design dataset using a) correct classification and b) correct cross-classification.** While red bars correspond to the expected percentage of correctly classified, respectively cross-classified calls, blue bars represent the mean percentage of correct classified and cross-classified calls obtained from real dataset**
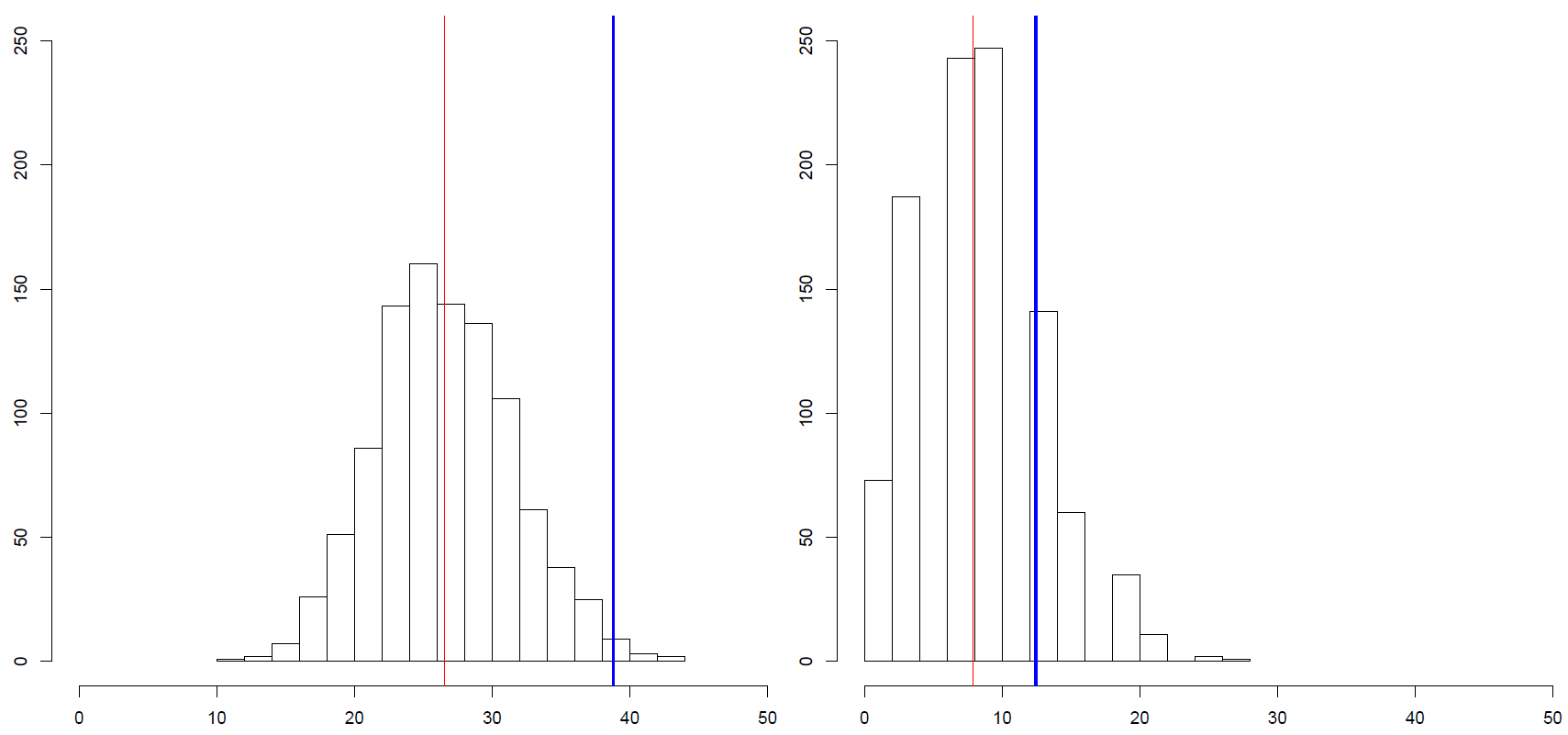
**.


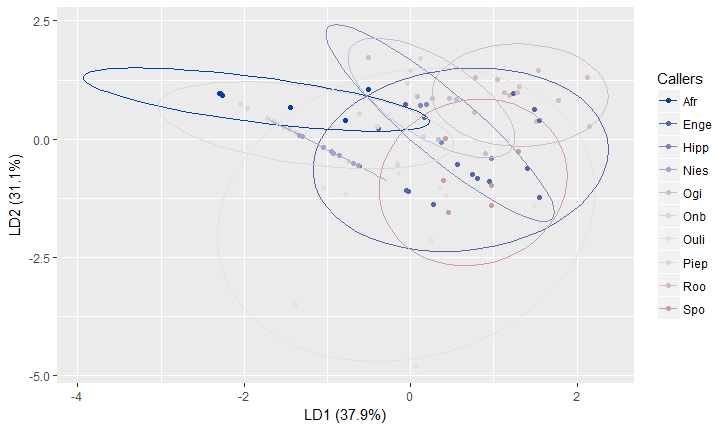


**Fig S15. Graph showing ﬁrst and second discriminant component scores resulting from an analysis of 85 bouts produced by 13 individuals, represented by the different colours.** Both axes represent the percentage of the variance explained by the ﬁrst and second discriminant functions, respectively.
