# Supplementary material for: Correlates of social role and conflict severity in wild vervet monkey agonistic screams
Source: PLoS One. 2019 May 1;14(5):e0214640. doi: 10.1371/journal.pone.0214640 (PMC6493722; doi:10.1371/journal.pone.0214640)
Supplement: S8 Appendix — (DOCX) [file pone.0214640.s008.docx]

S8. Detailed results of the analyses at the call level

1. Permutated Discriminant Function Analyses

We used 187 screams produced by 13 individuals to investigate whether contexts (screams recorded during natural follows vs around experiments) affected nine call related acoustic parameters: scream duration, peak frequency, coefficient of frequency variation, coefficient of frequency modulation, absolute transition onset, absolute transition offset, frequency quartile 50, inter-quartile range and Shannon entropy. However, we had to exclude the coefficient of frequency variation from further analyses due to its high correlation with Q50. Results from pDFAs showed that we were not able to discriminate screams according to the context of production (51.41% expected calls correctly cross-classified compared to 57.14% calls correctly cross-classified, *P* = 0.11). Similarly, we used 82 screams produced by four individuals to investigate whether callers’ identity affected the same acoustic parameters. Results from pDFAs showed that screams were individually distinctive (24.9% expected vs 47.2% calls correctly cross-classified, *P* = 0.002). To reproduce those two results, please visit Figshare (Mercier, Déaux et al. 2018).

1. Linear Mixed Models

As context did not affected our acoustic parameters in significant ways, we thus used screams produced in both contexts by all individuals for further analyses, leading to a dataset of 301 screams produced by 25 individuals. We performed 12 linear mixed models fitted by restricted maximum likelihood (REML) with Laplace approximation, normal or lognormal distributions and logit-link function (LMER; Bates, Mächler et al. 2015) for each acoustic parameter, using the later one as the response variable and three fixed effects: social role of signallers, conflict severity and their interaction. We included caller identity and events as random effects to control for repeated measures. We then checked for homogeneity of the data and the distribution of residuals using graphical analyses of residuals (using bwplots, qqplots and binned plots) and checked for influential individuals and outliers, removing them only when necessary (if it did help to reach approximate symmetrical distribution and did not affect our results). For more details, please see Figshare (Mercier, Déaux et al. 2018).

1. **Duration**

| REML criterion at convergence: 761.1  Number of observations: 301 Number of events: 234  Number of callers: 25 | | | | | |
| --- | --- | --- | --- | --- | --- |
| Scaled residuals | Min  -2.252 | 1Q  -0.652 | Median  0.011 | 3Q  0.726 | Max  2.47 |
| Random effects | Groups | Name | Variance | Std. Dev. |  |
|  | NbEvent | (Intercept) | 0.001 | 0.036 |  |
|  | Caller | (Intercept) | 0.016 | 0.127 |  |
|  | Residual |  | 0.702 | 0.838 |  |
| R squared values (R^2^m = marginal R^2^ explained by fixed effects only and R^2^c = conditional R^2^ explained by both fixed and random effects): | | | | | |
| R^2^m = 0.070 | | | | | |
| R^2^c = 0.093 | | | | | |

**Table S8. Results from the linear mixed model testing variation found in scream duration**

|  | Estimate | Std. Error | T value | CIL | CIU | *P* | Adjusted *P* |
| --- | --- | --- | --- | --- | --- | --- | --- |
| (Intercept) | -1.546 | 0.096 | -16.095 | -1.73 | -1.36 | NA | NA |
| Social role (Victim) | 0.512 | 0.131 | 3.915 | 0.26 | 0.77 | <0.001 | <0.001 |
| Severity (Severe) | 0.303 | 0.156 | 1.943 | 0 | 0.61 | 0.133 | 0.318 |
| Social role : Severity | -0.262 | 0.206 | -1.273 | -0.67 | 0.14 | 0.203 | 0.365 |

1. **Peak frequency**

| REML criterion at convergence: 1024.1  Number of observations: 301  Number of Events: 234  Number of callers: 25 | | | | | |
| --- | --- | --- | --- | --- | --- |
| Scaled residuals | Min  -3.0093 | 1Q  -0.4684 | Median  -0.0301 | 3Q  0.4144 | Max  3.7806 |
| Random effects | Groups | Name | Variance | Std. Dev. |  |
|  | NbEvent | (Intercept) | 0.2375 | 0.4874 |  |
|  | Caller | (Intercept) | 0.9298 | 0.9642 |  |
|  | Residual |  | 1.2640 | 1.1243 |  |
| R squared values (R^2^m = marginal R^2^ explained by fixed effects only and R^2^c = conditional R^2^ explained by both fixed and random effects): | | | | | |
| R^2^m = 0.008 | | | | | |
| R^2^c = 0.484 | | | | | |

**Table S9. Results from the linear mixed model testing variation found in peak frequency**

|  | Estimate | Std. Error | T value | CIL | CIU | *P* | Adjusted *P* |
| --- | --- | --- | --- | --- | --- | --- | --- |
| (Intercept) | 5.204 | 0.244 | 21.329 | 4.73 | 5.68 | NA | NA |
| Social role (Victim) | -0.087 | 0.203 | -0.426 | -0.48 | 0.31 | 0.652 | 0.734 |
| Severity (Severe) | -0.276 | 0.252 | -1.094 | -0.77 | 0.22 | 0.130 | 0.318 |
| Social role : Severity | 0.04 | 0.331 | 0.121 | -0.61 | 0.69 | 0.904 | 0.904 |

1. **Coefficient of frequency modulation**

| REML criterion at convergence: 870  Number of observations: 300 (1 outlier removed) Number of events: 234  Number of callers: 25 | | | | | |
| --- | --- | --- | --- | --- | --- |
| Scaled residuals | Min  -2.3752 | 1Q  -0.4327 | Median  -0.0035 | 3Q  0.4414 | Max  2.6367 |
| Random effects | Groups | Name | Variance | Std. Dev. |  |
|  | NbEvent | (Intercept) | 0.4832 | 0.6952 |  |
|  | Caller | (Intercept) | 0.2949 | 0.5430 |  |
|  | Residual |  | 0.5256 | 0.7250 |  |
| R squared values (R^2^m = marginal R^2^ explained by fixed effects only and R^2^c = conditional R^2^ explained by both fixed and random effects): | | | | | |
| R^2^m = 0.010 | | | | | |
| R^2^c = 0.601 | | | | | |

**Table S10. Results from the linear mixed model testing variation found in coefficient of frequency modulation**

|  | Estimate | Std. Error | T value | CIL | CIU | *P* | Adjusted *P* |
| --- | --- | --- | --- | --- | --- | --- | --- |
| (Intercept) | 1.782 | 0.161 | 11.039 | 1.47 | 2.1 | NA | NA |
| Social role (Victim) | 0.022 | 0.162 | 0.136 | -0.3 | 0.34 | 0.359 | 0.538 |
| Severity (Severe) | 0.296 | 0.206 | 1.438 | -0.11 | 0.7 | 0.548 | 0.707 |
| Social role : Severity | -0.369 | 0.267 | -1.38 | -0.89 | 0.15 | 0.168 | 0.336 |

1. **Absolute transition onset**

| REML criterion at convergence: 859.8  Number of observations: 301  Number of events: 234  Number of callers: 25 | | | | | |
| --- | --- | --- | --- | --- | --- |
| Scaled residuals | Min  -2.1422 | 1Q  -0.6646 | Median  -0.0611 | 3Q  0.7802 | Max  1.9251 |
| Random effects | Groups | Name | Variance | Std. Dev. |  |
|  | NbEvent | (Intercept) | 0.0986 | 0.3140 |  |
|  | Caller | (Intercept) | 0.0798 | 0.2824 |  |
|  | Residual |  | 0.8560 | 0.9252 |  |
| R squared values (R^2^m = marginal R^2^ explained by fixed effects only and R^2^c = conditional R^2^ explained by both fixed and random effects): | | | | | |
| R^2^m = 0.014 | | | | | |
| R^2^c = 0.184 | | | | | |

**Table S11. Results from the linear mixed model testing variation found in absolute transition onset**

|  | Estimate | Std. Error | T value | CIL | CIU | *P* | Adjusted *P* |
| --- | --- | --- | --- | --- | --- | --- | --- |
| (Intercept) | 0.312 | 0.126 | 2.474 | 0.06 | 0.56 | NA | NA |
| Social role (Victim) | -0.252 | 0.157 | -1.607 | -0.56 | 0.06 | 0.148 | 0.318 |
| Severity (Severe) | 0.054 | 0.19 | 0.286 | -0.32 | 0.43 | 0.184 | 0.349 |
| Social role : Severity | 0.193 | 0.251 | 0.769 | -0.3 | 0.68 | 0.442 | 0.636 |

1. **Absolute transition offset**

| REML criterion at convergence: 841.2  Number of observations: 300 (1 outlier removed) Number of events: 234  Number of callers: 25 | | | | | |
| --- | --- | --- | --- | --- | --- |
| Scaled residuals | Min  -2.1475 | 1Q  -0.7919 | Median  -0.0361 | 3Q  0.7179 | Max  2.1666 |
| Random effects | Groups | Name | Variance | Std. Dev. |  |
|  | NbEvent | (Intercept) | 0.0000 | 0.0000 |  |
|  | Caller | (Intercept) | 0.1203 | 0.3468 |  |
|  | Residual |  | 0.8821 | 0.9392 |  |
| R squared values (R^2^m = marginal R^2^ explained by fixed effects only and R^2^c = conditional R^2^ explained by both fixed and random effects): | | | | | |
| R^2^m = 0.018 | | | | | |
| R^2^c = 0.136 | | | | | |

**Table S12. Results from the linear mixed model testing variation found in absolute transition offset**

|  | Estimate | | Std. Error | | T value | CIL | | CIU | *P* | | Adjusted *P* | |
| --- | --- | --- | --- | --- | --- | --- | --- | --- | --- | --- | --- | --- |
| (Intercept) | | 0.145 | | 0.129 | 1.118 | | -0.11 | 0.4 | | NA | NA |  |
| Social role (Victim) | | -0.151 | | 0.152 | -0.991 | | -0.45 | 0.15 | | 0.312 | 0.493 |  |
| Severity (Severe) | | 0.218 | | 0.183 | 1.189 | | -0.14 | 0.58 | | 0.028 | 0.112 |  |
| Social role : Severity | | 0.081 | | 0.243 | 0.334 | | -0.4 | 0.56 | | 0.739 | 0.806 |  |

1. **Frequency quartile 50**

| REML criterion at convergence: -0.3  Number of observations: 293  Number of events: 228  Number of callers: 24 (1 influential individual removed) | | | | | |
| --- | --- | --- | --- | --- | --- |
| Scaled residuals | Min  -2.4583 | 1Q  -0.5534 | Median  -0.0391 | 3Q  0.5550 | Max  2.8613 |
| Random effects | Groups | Name | Variance | Std. Dev. |  |
|  | NbEvent | (Intercept) | 0.0083 | 0.0909 |  |
|  | Caller | (Intercept) | 0.0073 | 0.0854 |  |
|  | Residual |  | 0.0438 | 0.2092 |  |
| R squared values (R^2^m = marginal R^2^ explained by fixed effects only and R^2^c = conditional R^2^ explained by both fixed and random effects): | | | | | |
| R^2^m = 0.048 | | | | | |
| R^2^c = 0.298 | | | | | |

**Table S13. Results from the linear mixed model testing variation found in frequency quartile 50**

|  | Estimate | Std. Error | T value | CIL | CIU | *P* | | Adjusted *P* |  |
| --- | --- | --- | --- | --- | --- | --- | --- | --- | --- |
| (Intercept) | 0.451 | 0.033 | 13.851 | 0.39 | 0.51 | | NA | NA | |
| Social role (Victim) | 0.068 | 0.038 | 1.813 | -0.01 | 0.14 | | 0.003 | 0.022 | |
| Severity (Severe) | 0.03 | 0.046 | 0.669 | -0.06 | 0.12 | | 0.068 | 0.222 | |
| Social role : Severity | 0.043 | 0.06 | 0.71 | -0.08 | 0.16 | | 0.478 | 0.662 | |

1. **Inter-quartile range**

| REML criterion at convergence: -249.4  Number of observations: 301  Number of events: 234  Number of callers: 25 | | | | | |
| --- | --- | --- | --- | --- | --- |
| Scaled residuals | Min  -2.6477 | 1Q  -0.6356 | Median  -0.0203 | 3Q  0.5679 | Max  2.9425 |
| Random effects | Groups | Name | Variance | Std. Dev. |  |
|  | NbEvent | (Intercept) | 0.0017 | 0.0415 |  |
|  | Caller | (Intercept) | 0.0034 | 0.0585 |  |
|  | Residual |  | 0.0205 | 0.1430 |  |
| R squared values (R^2^m = marginal R^2^ explained by fixed effects only and R^2^c = conditional R^2^ explained by both fixed and random effects): | | | | | |
| R^2^m = 0.005 | | | | | |
| R^2^c = 0.205 | | | | | |

**Table S14. Results from the linear mixed model testing variation found in inter-quartile range**

|  | Estimate | Std. Error | T value | CIL | CIU | *P* | Adjusted *P* |
| --- | --- | --- | --- | --- | --- | --- | --- |
| (Intercept) | 0.405 | 0.021 | 19.257 | 0.36 | 0.45 | NA | NA |
| Social role (Victim) | 0.025 | 0.024 | 1.054 | -0.02 | 0.07 | 0.589 | 0.707 |
| Severity (Severe) | 0.011 | 0.029 | 0.387 | -0.05 | 0.07 | 0.572 | 0.707 |
| Social role : Severity | -0.039 | 0.039 | -1.004 | -0.12 | 0.04 | 0.315 | 0.493 |

1. **Shannon entropy**

| REML criterion at convergence: -475.8  Number of observations: 300 (1 outlier removed) Number of events: 234  Number of callers: 25 | | | | | |
| --- | --- | --- | --- | --- | --- |
| Scaled residuals | Min  -3.2005 | 1Q  -0.5877 | Median  0.1123 | 3Q  0.6541 | Max  2.1220 |
| Random effects | Groups | Name | Variance | Std. Dev. |  |
|  | NbEvent | (Intercept) | <0.001 | <0.001 |  |
|  | Caller | (Intercept) | 0.0031 | 0.0556 |  |
|  | Residual |  | 0.0099 | 0.0993 |  |
| R squared values (R^2^m = marginal R^2^ explained by fixed effects only and R^2^c = conditional R^2^ explained by both fixed and random effects): | | | | | |
| R^2^m = 0.103 | | | | | |
| R^2^c = 0.317 | | | | | |

**Table S15. Results from the linear mixed model testing variation found in Shannon entropy**

|  | Estimate | Std. Error | T value | CIL | CIU | *P* | Adjusted *P* |
| --- | --- | --- | --- | --- | --- | --- | --- |
| (Intercept) | 1.132 | 0.016 | 69.568 | 1.1 | 1.16 | NA | NA |
| Social role (Victim) | 0.008 | 0.016 | 0.503 | -0.02 | 0.04 | 0.022 | 0.099 |
| Severity (Severe) | 0.033 | 0.02 | 1.671 | -0.01 | 0.07 | <0.001 | <0.001 |
| Social role : Severity | 0.052 | 0.026 | 1.961 | 0 | 0.1 | 0.050 | 0.180 |


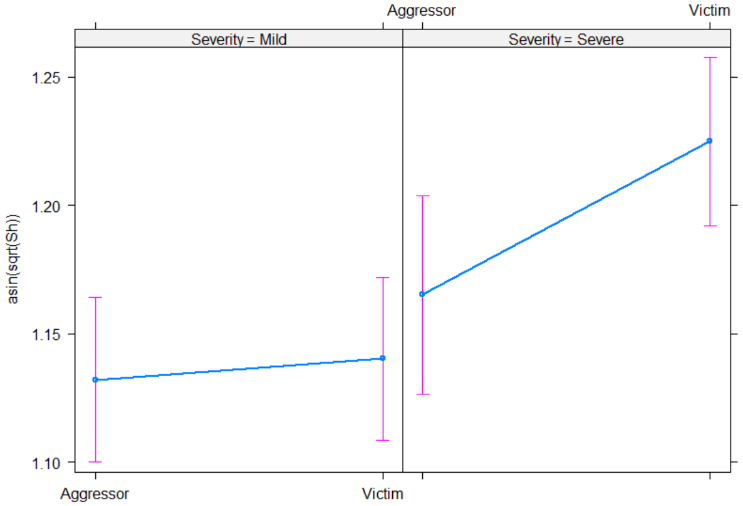

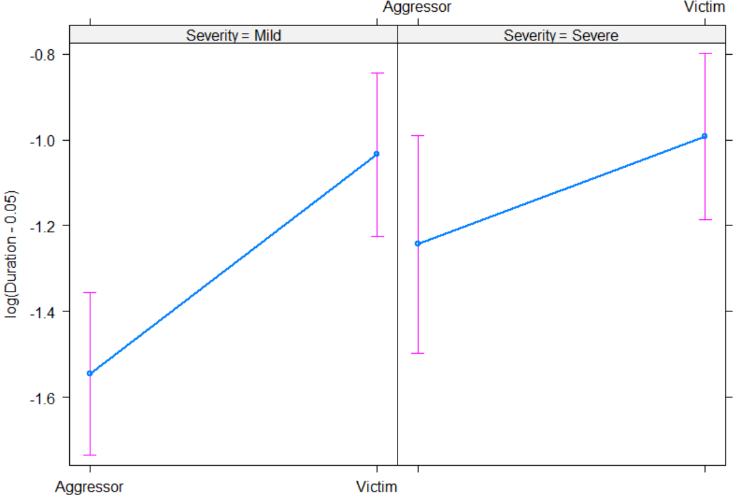


a)


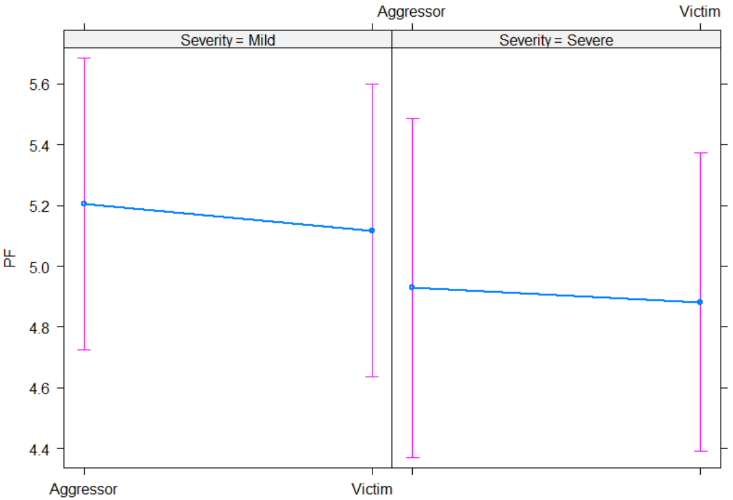


b)


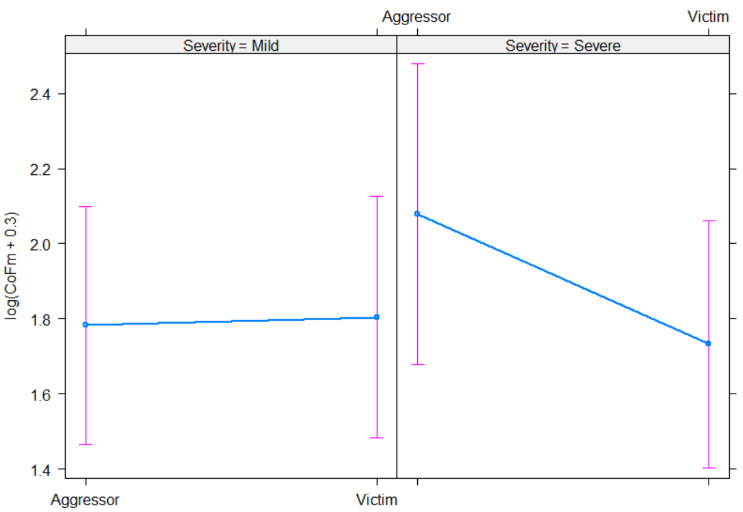


c)


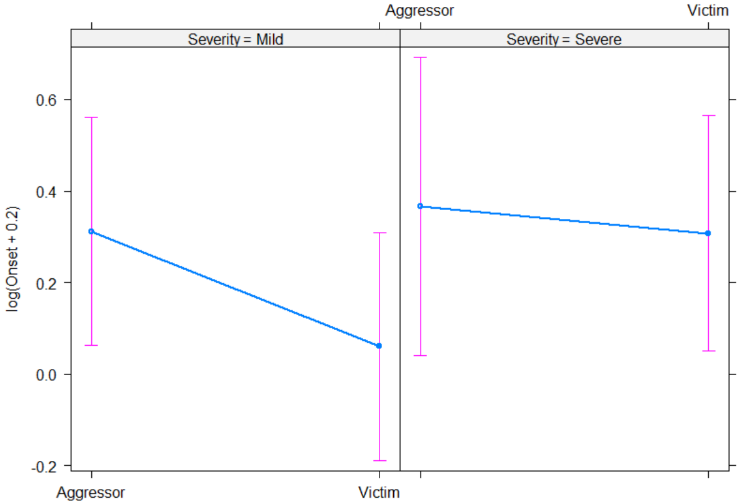


d)


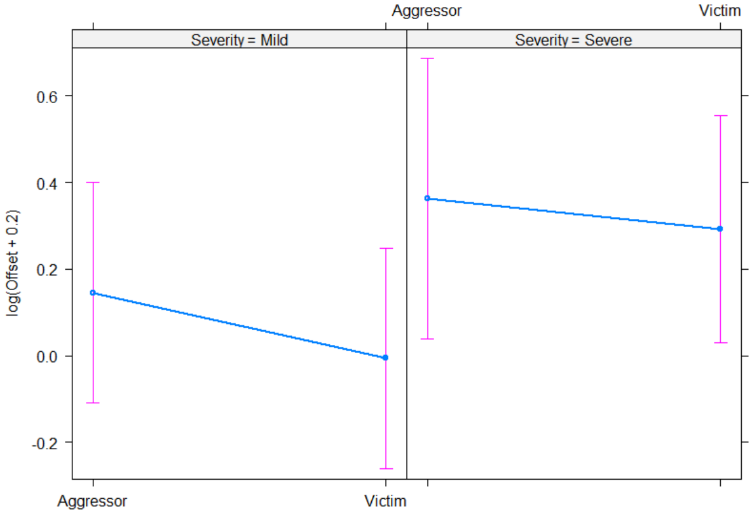


e)


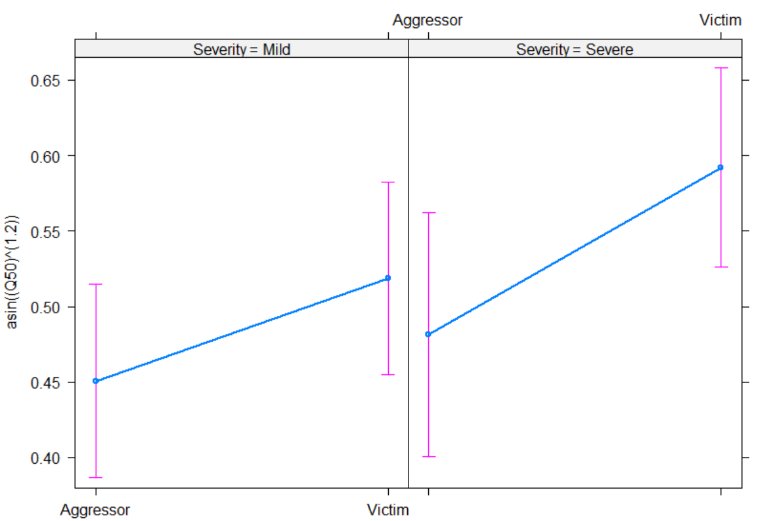


f)


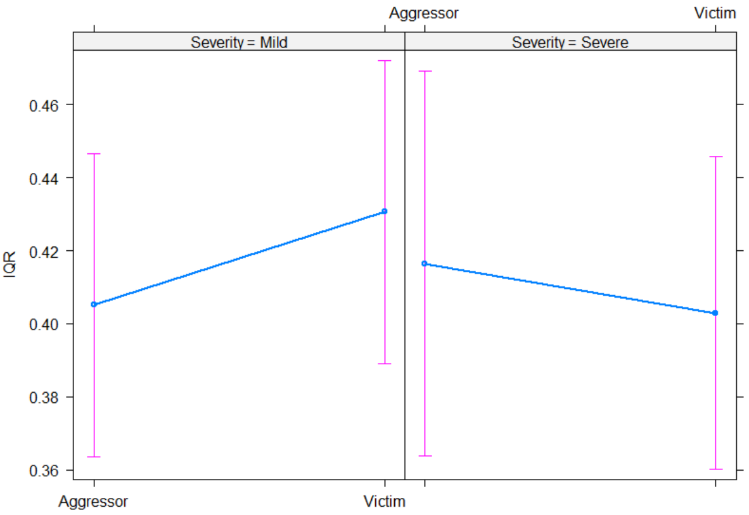


g)

h)

**Figure S16. Effect plot showing the influences of both social role and severity on eight acoustic parameters tested at the call level:** a) scream duration, b) peak frequency, c) coefficient of frequency modulation, d) absolute transition onset, e) absolute transition offset, f) frequency quartile 50, g) inter-quartile range and h) Shannon entropy.
