# Supplementary material for: Correlates of social role and conflict severity in wild vervet monkey agonistic screams
Source: PLoS One. 2019 May 1;14(5):e0214640. doi: 10.1371/journal.pone.0214640 (PMC6493722; doi:10.1371/journal.pone.0214640)
Supplement: S9 Appendix — (DOCX) [file pone.0214640.s009.docx]

S9. Detailed results of the analyses at the bout level

1. Permutated Discriminant Function Analyses

We used 102 bouts of screams produced by 15 individuals to investigate whether contexts (screams recorded during natural follows vs around experiments) affected six bout related acoustic parameters: bout duration, number of screams, average scream duration, scream intervals, scream rate and percentage of screams with NLP. However, we had to exclude bout duration from further analyses due to its high correlation with the number of screams and scream rate. Furthermore, we removed scream intervals, as we did not reach approximate symmetrical distribution, thus violating the assumption of normality. Results from pDFAs showed that we were not able to discriminate bouts according to the context of production (50.5% expected calls correctly cross-classified compared to 41.9% calls correctly cross-classified, *P* = 0.88). Similarly, we used 61 bouts of screams produced by eight individuals to investigate whether callers’ identity affected the same acoustic parameters. Results from pDFAs showed that we were not able to discriminate bouts according to individuals (36.1% expected vs 44.1 % calls correctly cross-classified, *P* = 0.1). To reproduce those two results, please visit Figshare (Mercier, Déaux et al. 2018).

1. Linear Mixed Models

As context did not affect our acoustic parameters in significant ways, we thus used bouts of screams produced in both contexts for further analyses, leading to a total dataset of 137 bouts produced by 26 individuals. We performed four linear mixed models fitted by restricted maximum likelihood (REML) with Laplace approximation, normal or lognormal distributions and logit-link function (LMER; Bates, Mächler et al. 2015) for each acoustic parameter, using the later one as the response variable and three fixed effects: social role of signallers, conflict severity and their interaction. We included caller identity as a random effect to control for repeated measures. We then checked for homogeneity of the data and the distribution of residuals using graphical analyses of residuals (using bwplots, qqplots and binned plots) and checked for influential individuals and outliers, removing them only when necessary (if it did help to reach approximate symmetrical distribution and did not affect our results). For more details, please see Figshare (Mercier, Déaux et al. 2018).

1. **Number of screams**

| REML criterion at convergence: 320.4  Number of observations: 117 (1 outlier removed)  Number of callers: 26 | | | | | |
| --- | --- | --- | --- | --- | --- |
| Scaled residuals | Min  -1.7018 | 1Q  -0.9497 | Median  0.1196 | 3Q  0.6637 | Max  2.0314 |
| Random effects | Groups | Name | Variance | Std. Dev. |  |
|  | Caller | (Intercept) | 0.2367 | 0.4865 |  |
|  | Residual |  | 0.7373 | 0.8586 |  |
| R squared values (R^2^m = marginal R^2^ explained by fixed effects only and R^2^c = conditional R^2^ explained by both fixed and random effects): | | | | | |
| R^2^m = 0.022 | | | | | |
| R^2^c = 0.259 | | | | | |

**Table S16. Results from the linear mixed model testing variation found in the number of screams**

|  | Estimate | Std. Error | T value | CIL | CIU | *P* | Adjusted *P* |
| --- | --- | --- | --- | --- | --- | --- | --- |
| (Intercept) | 0.9543 | 0.1848 | 5.1635 | 0.59 | 1.32 | NA | NA |
| Social role (Victim) | 0.016 | 0.2144 | 0.0748 | -0.4 | 0.44 | 0.312 | 0.493 |
| Severity (Severe) | 0.3551 | 0.2731 | 1.3004 | -0.18 | 0.89 | 0.773 | 0.818 |
| Social role : Severity | -0.4995 | 0.3465 | -1.4413 | -1.18 | 0.18 | 0.150 | 0.318 |

1. **Average scream duration**

| REML criterion at convergence: 248.1  Number of observations: 118  Number of callers: 26 | | | | | |
| --- | --- | --- | --- | --- | --- |
| Scaled residuals | Min  -2.4804 | 1Q  -0.6586 | Median  -0.0025 | 3Q  0.6469 | Max  2.7525 |
| Random effects | Groups | Name | Variance | Std. Dev. |  |
|  | Caller | (Intercept) | 0.0261 | 0.1615 |  |
|  | Residual |  | 0.4368 | 0.6609 |  |
| R squared values (R^2^m = marginal R^2^ explained by fixed effects only and R^2^c = conditional R^2^ explained by both fixed and random effects): | | | | | |
| R^2^m = 0.140 | | | | | |
| R^2^c = 0.188 | | | | | |

**Table S17. Results from the linear mixed model testing variation found in average scream duration**

|  | Estimate | Std. Error | T value | CIL | CIU | *P* | Adjusted *P* |
| --- | --- | --- | --- | --- | --- | --- | --- |
| (Intercept) | -1.3302 | 0.1217 | -10.93 | -1.57 | -1.09 | NA | NA |
| Social role (Victim) | 0.3734 | 0.1608 | 2.3213 | 0.06 | 0.69 | 0.010 | 0.051 |
| Severity (Severe) | 0.4805 | 0.2043 | 2.3519 | 0.08 | 0.88 | 0.002 | 0.018 |
| Social role : Severity | -0.1254 | 0.2601 | -0.4823 | -0.64 | 0.38 | 0.630 | 0.732 |

1. **Scream rate**

| REML criterion at convergence: 308.5  Number of observations: 118  Number of callers: 26 | | | | | |
| --- | --- | --- | --- | --- | --- |
| Scaled residuals | Min  -3.1794 | 1Q  -0.5854 | Median  -0.0419 | 3Q  0.5221 | Max  2.4408 |
| Random effects | Groups | Name | Variance | Std. Dev. |  |
|  | Caller | (Intercept) | 0.0944 | 0.3073 |  |
|  | Residual |  | 0.7074 | 0.8411 |  |
| R squared values (R^2^m = marginal R^2^ explained by fixed effects only and R^2^c = conditional R^2^ explained by both fixed and random effects): | | | | | |
| R^2^m = 0.088 | | | | | |
| R^2^c = 0.196 | | | | | |

**Table S18. Results from the linear mixed model testing variation found in scream rate**

|  | Estimate | Std. Error | T value | CIL | CIU | *P* | Adjusted *P* |
| --- | --- | --- | --- | --- | --- | --- | --- |
| (Intercept) | 0.3676 | 0.1634 | 2.2494 | 0.05 | 0.69 | NA | NA |
| Social role (Victim) | 0.1082 | 0.207 | 0.5224 | -0.3 | 0.51 | 0.074 | 0.222 |
| Severity (Severe) | -0.763 | 0.263 | -2.9011 | -1.28 | -0.25 | 0.005 | 0.030 |
| Social role : Severity | 0.4849 | 0.334 | 1.4516 | -0.17 | 1.14 | 0.147 | 0.318 |

1. **NLP**

| REML criterion at convergence: 109.5  Number of observations: 116 (2 outliers removed)  Number of callers: 26 | | | | | |
| --- | --- | --- | --- | --- | --- |
| Scaled residuals | Min  -2.7990 | 1Q  -0.5608 | Median  0.1522 | 3Q  0.7823 | Max  1.8681 |
| Random effects | Groups | Name | Variance | Std. Dev. |  |
|  | Caller | (Intercept) | 0.0364 | 0.1907 |  |
|  | Residual |  | 0.1153 | 0.3396 |  |
| R squared values (R^2^m = marginal R^2^ explained by fixed effects only and R^2^c = conditional R^2^ explained by both fixed and random effects): | | | | | |
| R^2^m = 0.131 | | | | | |
| R^2^c = 0.339 | | | | | |

**Table S19. Results from the linear mixed model testing variation found in NLP**

|  | Estimate | Std. Error | T value | CIL | CIU | *P* | Adjusted *P* |
| --- | --- | --- | --- | --- | --- | --- | --- |
| (Intercept) | 1.1587 | 0.0741 | 15.633 | 1.01 | 1.3 | NA | NA |
| Social role (Victim) | -0.014 | 0.0861 | -0.1628 | -0.18 | 0.15 | 0.800 | 0.823 |
| Severity (Severe) | 0.2506 | 0.1084 | 2.311 | 0.04 | 0.46 | <0.001 | <0.001 |
| Social role : Severity | 0.0805 | 0.1373 | 0.5864 | -0.19 | 0.35 | 0.558 | 0.707 |


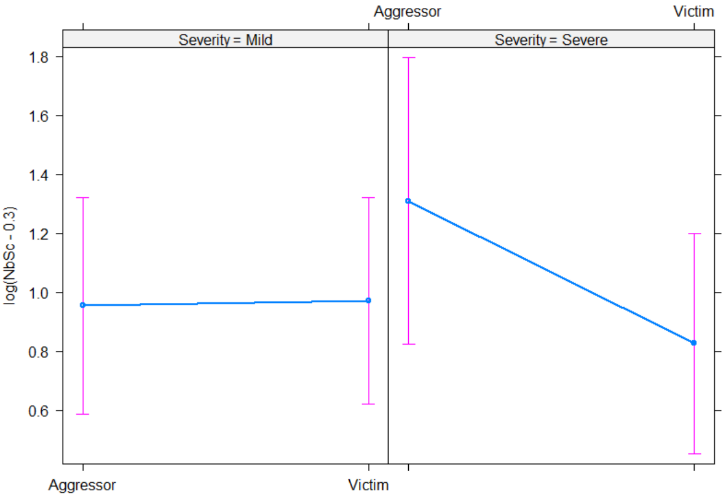


a)


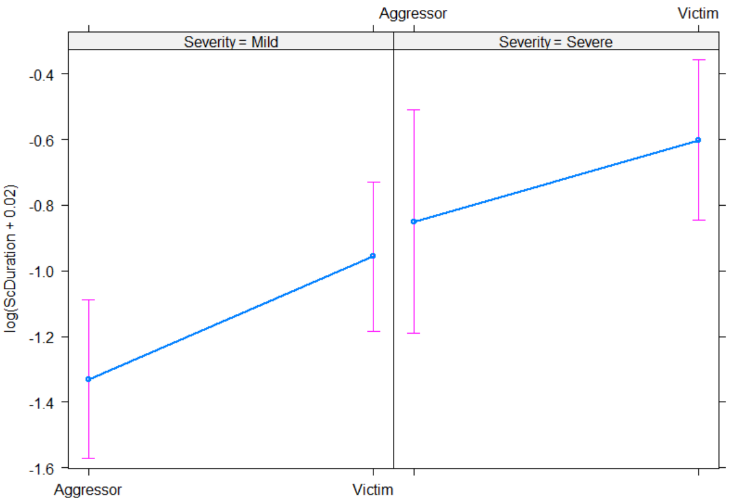


b)


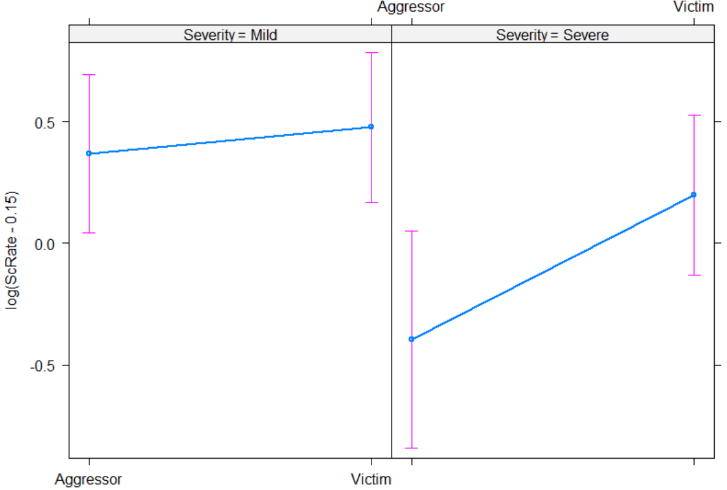


c)


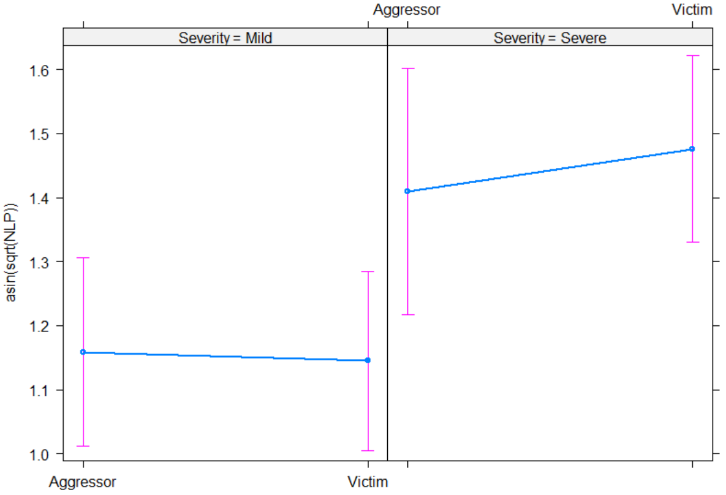


d)

**Fig S17. Effect plot showing the influences of both social role and severity on four acoustic parameters at the bout level:** a) number of screams, b) average scream duration, c) scream rate and d) percentage of non-linear phenomena.
