# Supplementary material for: Correlates of social role and conflict severity in wild vervet monkey agonistic screams
Source: PLoS One. 2019 May 1;14(5):e0214640. doi: 10.1371/journal.pone.0214640 (PMC6493722; doi:10.1371/journal.pone.0214640)
Supplement: S10 Appendix — (DOCX) [file pone.0214640.s010.docx]

S10. Detailed results of the support analysis

Linear Mixed Models

We used 111 bouts of screams produced by 26 individuals to perform a generalized linear mixed model [GLMM; 1] fitted with a binomial structure and logit-link function to examine the influence of the social role of signallers (aggressor vs. victim) and conflict severity (mild vs. severe) on the number of support received. We used the later one as the response variable and three fixed effects: social role of signallers, conflict severity and their interaction. We included both caller identity and context of production (using six levels: observation, observation without food, observation with food involved, experiments, experiments without food and experiments with food involved) as random effects to control for repeated measures, thus avoiding pseudo-replication [2]. After checking for multicollinearity (all r < 0.80), we then checked for homogeneity of the data and the distribution of residuals using graphical analyses of residuals (using bwplots, qqplots and binned plots) and checked for influential individuals and outliers, not removing any of them. For more details, please see Figshare [3].

1. **Number of support**

| AIC BIC logLik deviance df.resid  90.6 106.8 -39.3 78.6 105  Number of observations: 111  Number of callers: 26 | | | | | |
| --- | --- | --- | --- | --- | --- |
| Scaled residuals | Min  -1.3798 | 1Q  -0.3601 | Median  -0.1762 | 3Q  -0.1234 | Max  4.8223 |
| Random effects | Groups | Name | Variance | Std. Dev. |  |
|  | Caller | (Intercept) | 1.081 | 1.04 |  |
|  | Context | (Intercept) | 0.000 | 0.00 |  |
| R squared values (R^2^m = marginal R^2^ explained by fixed effects only and R^2^c = conditional R^2^ explained by both fixed and random effects): | | | | | |
| R^2^m = 0.388 | | | | | |
| R^2^c = 0.540 | | | | | |

**Table S20. Results from the GLMM testing variation found in the number of support**

|  | Estimate | Std. Error | Z value | CIL | CIU | *P* |
| --- | --- | --- | --- | --- | --- | --- |
| (Intercept) | -1.401 | 0.610 | -2.298 | -2.595 | -0.206 | NA |
| Social role (Victim) | -1.860 | 0.907 | -2.050 | -3.638 | -0.082 | 0.040 |
| Severity (Severe) | 2.559 | 1.053 | 2.430 | 0.495 | 4.623 | 0.015 |
| Social role : Severity | -3.035 | 1.679 | -1.807 | -6.326 | 0.256 | 0.071 |
